# EPAS1 Gain-of-Function Mutation Contributes to High-Altitude Adaptation in Tibetan Horses

**DOI:** 10.1093/molbev/msz158

**Published:** 2019-07-04

**Authors:** Xuexue Liu, Yanli Zhang, Yefang Li, Jianfei Pan, Dandan Wang, Weihuang Chen, Zhuqing Zheng, Xiaohong He, Qianjun Zhao, Yabin Pu, Weijun Guan, Jianlin Han, Ludovic Orlando, Yuehui Ma, Lin Jiang

**Affiliations:** 1 Laboratory of Animal (Poultry) Genetics Breeding and Reproduction, Ministry of Agriculture, Institute of Animal Science, Chinese Academy of Agricultural Sciences (CAAS), Beijing, PR China; 2 CAAS-ILRI Joint Laboratory on Livestock and Forage Genetic Resources, Institute of Animal Science, Chinese Academy of Agricultural Sciences (CAAS), Beijing, PR China; 3 College of Animal Science and Technology, Northwest A&F University, Yangling, Shaanxi, PR China; 4 International Livestock Research Institute (ILRI), Nairobi, Kenya; 5 Lundbeck Foundation GeoGenetics Center, University of Copenhagen, Denmark; 6 Laboratoire d'Anthropobiologie Moléculaire et d'Imagerie de Synthèse, CNRS, UMR 5288, Université Paul Sabatier (UPS), Toulouse, France

**Keywords:** Tibetan horse, hypoxia adaptation, EPAS1, respiration, metabolism, convergence

## Abstract

High altitude represents some of the most extreme environments worldwide. The genetic changes underlying adaptation to such environments have been recently identified in multiple animals but remain unknown in horses. Here, we sequence the complete genome of 138 domestic horses encompassing a whole altitudinal range across China to uncover the genetic basis for adaptation to high-altitude hypoxia. Our genome data set includes 65 lowland animals across ten Chinese native breeds, 61 horses living at least 3,300 m above sea level across seven locations along Qinghai-Tibetan Plateau, as well as 7 Thoroughbred and 5 Przewalski’s horses added for comparison. We find that Tibetan horses do not descend from Przewalski’s horses but were most likely introduced from a distinct horse lineage, following the emergence of pastoral nomadism in Northwestern China ∼3,700 years ago. We identify that the *endothelial PAS domain protein 1* gene (*EPAS1*, also *HIF2A*) shows the strongest signature for positive selection in the Tibetan horse genome. Two missense mutations at this locus appear strongly associated with blood physiological parameters facilitating blood circulation as well as oxygen transportation and consumption in hypoxic conditions. Functional validation through protein mutagenesis shows that these mutations increase EPAS1 stability and its hetero dimerization affinity to ARNT (HIF1B). Our study demonstrates that missense mutations in the *EPAS1* gene provided key evolutionary molecular adaptation to Tibetan horses living in high-altitude hypoxic environments. It reveals possible targets for genomic selection programs aimed at increasing hypoxia tolerance in livestock and provides a textbook example of evolutionary convergence across independent mammal lineages.

## Introduction

Hypoxia and ultraviolet exposure are key characteristics of high-altitude environments. Recent functional genomic studies have revealed the genetic basis of adaption to high-altitude hypoxia in Tibetan people (Simonson et al. 2010; Yi et al. 2014), Tibetan dogs (Wang et al. 2014), antelopes (Ge et al. 2013) and gray wolves (Zhang et al. 2014). Although other selection targets have been reported, the *endothelial PAS domain protein 1* (*EPAS1*) gene often appears as a common selection target, providing a striking example of convergent evolution across a range of mammals exposed to similar environmental pressure in the Qinghai-Tibetan (QT) Plateau. The *EPAS1* gene encodes one subunit of the hypoxia-inducible factor (HIF) and shows multifarious effects, including the regulation of angiogenesis, hemoglobin concentration (HMG) and erythrocytosis (Beall et al. 2010). Other mechanisms, such as the higher levels of nitric oxide (NO) enhancing the pulmonary blood flow and oxygen delivery (Hoit et al. 2005), are also known to facilitate life at high altitude. Additionally, other convergent modifications of metabolic pathways and the composition of rumen microbiota (Zhang et al. 2016) have been described in Tibetan Sherpas (Horscroft et al. 2017), the yak (Qiu et al. 2012), and other high-altitude mammals (Ge et al. 2013).

Many livestock species other than the dogs and the yak live in the QT Plateau (above 3,000 m), including chicken (Wang et al. 2015), sheep (Wei et al. 2016), and goats (Song et al. 2016). They are all known to exhibit higher hemoglobin levels than their lowland relatives. Domestic horses have also inhabited the QT Plateau for thousand years where they have significantly contributed to the development of human societies (Chen et al. 2015). The biological basis for their adaptation to life at high altitude has, however, not yet been studied.

In contrast to Tibetan horses, previous work has studied the adaptive response to high altitude in Andean horses and identified selection signatures within a genomic block encompassing the *EPAS1* gene (Hendrickson 2013). However, the density of the genotyping data collected was limited to 50 k SNPs, and the causative mutation(s) underlying horse adaptation to life at high altitude in the Andes could not be identified.

In this study, we undertook the first genomic analysis of selection signatures in Tibetan horses, aiming at identifying the genetic basis for adaptation to high-altitude hypoxia in the QT Plateau. To achieve this, we sequenced the genome of 138 Chinese horses spanning a wide range of altitudes, from the lowland regions of the Northeastern Plain to the mountains of the QT Plateau. We identified that the *EPAS1* gene and the *hemoglobin subunit epsilon 1* (*HBE1*) gene showed some of the strongest signatures of positive selection in the Tibetan horse genome. Further exploration of *EPAS1*genotypes in a larger panel of 908 horses and functional validation assays suggested that at least one missense mutation in one of the PAS domains is a key driver of the physiological response of Tibetan horses to high-altitude hypoxia.

## Results

### Genomic Variation

**Fig. 1.**
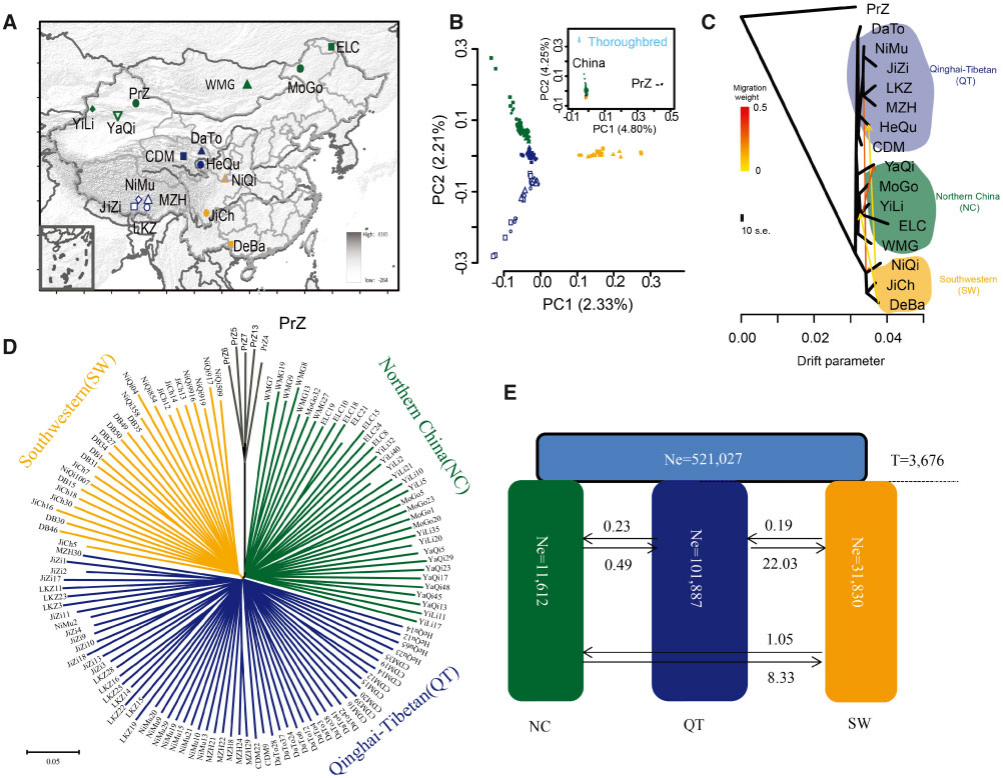
Geographic distribution, genetic structure and evolutionary relationships of Chinese native horse breeds. (*A*) The geographic distribution of 15 Chinese native horse populations and one Przewalski’s horse population (PrZ, black color). The blue color (hollow shapes) represents Tibetan horses (JiZi, Jiangzi; LKZ, Langkazi; MZH, Mozhu; NiMu, Nimu) living at least 4,000 m above the sea level (m.a.s.l.) whereas the blue color (solid shapes) represents Qinghai horses (CDM, Chaidamu; HeQu, Hequ; DaTo, Datong) living at an altitude of at least 3,000 m. The orange color represents the Southwestern horses (DeBa, Debao pony from Guangxi; JiCh, Jianchang pony from Sichuan; NiQi, Ningqiang pony from Shannxi). The green represents the Northern horses (MoGo, Mongolian horse from Inner Mongolia; WMG, Mongolian horse from Mongolia; ELC, ErlunChun horse from Heilongjiang; YaQi, Yanqi horse from Xinjiang; Yili horse from Xinjiang). The colors of symbols that indicate the geographic regions are the same as those in the PCA plots and phylogenetic trees. (*B*) PCA plots of the first two components of all horse samples (inner plot) and all Chinese native horses (outer plot). The fraction of the total variance explained is reported on each individual axis between parentheses. (*C*) The ML-TreeMix tree of all horses, with PrZ as outgroup, assuming four migration events. Four migration events that are most consistent with known events, because they increased the explained variance to 99.8% (Pickrell and Pritchard 2012), and does not increase afterwards (supplementary table S25, Supplementary Material online). Migration arrows are colored according to their weights. Horizontal branch lengths are proportional to the amount of genetic drift parameter that has occurred on the branch. The drift parameter measures the variance in allele frequency estimated along each branch of the tree. The yellow and orange lines indicate the instantaneous admixtures, whereas arrows denote continuous (unidirectional) gene flow. (*D*) The Neighbor-Joining tree of the horse breeds, with PrZ as outgroup. Bootstrap reported was close to 100%. (*E*) ∂a∂i best-supported population model depicting the evolutionary trajectories of the main three clusters of Chinese native horses. The light blue, green, dark blue and orange rectangles represent the ancestral, Northern China (NC), Qinghai-Tibetan (QT) and Southwestern (SW) populations, respectively. The numbers within the rectangles represent the effective size (individual horses) for the corresponding population. The average number of migrants per year between the different groups is shown between the black arrows. Ne = effective size (individuals). *T* = time of divergence (years).

We used Illumina HiSeq instruments to generate whole-genome sequencing data of 138 horses sampled from China (supplementary table S1, Supplementary Material online and fig. 1*A*). This provided a total of 25.87 billion of read pairs across Chinese domestic breeds (5–10 individuals per breed). Our data set also included seven Thoroughbreds and five Przewalski’s horses for comparison. The median genome coverage achieved across the full data set was ∼7.5X (min = 3.1X, max = 28.7X), representing ∼96.9% base coverage per individual genome (min = 66.7%, max = 98.4%, supplementary table S2, Supplementary Material online). Strict read alignment and genotyping calling procedures allowed us to identify a total of 10,376,152 single nucleotide polymorphisms (SNPs; supplementary fig. S1, Supplementary Material online), ∼46.0% of which were previously described in dbSNP (supplementary table S4, Supplementary Material online). Less than 1% of the SNP variants were located in exonic regions (30,094 [0.279%] were missense, 57,270 [0.456%] synonymous) (supplementary table S3, Supplementary Material online). Approximately 10% were present in downstream or upstream gene regulatory regions whereas the remaining dominant fraction (∼89%), spread across intergenic and intronic regions. We also identified 2,306,242 indels and 44,724 copy number variations (CNVs), comprising a total of 472.98 Mb (∼18%) of the equine genome (supplementary fig. S1, Supplementary Material online). Chinese native horses were found to show higher genome-wide heterozygosity levels, shorter runs of homozygosity (ROHs) and lower linkage disequilibrium (LD) decay than Przewalski’s horses and Thoroughbred racing horses (supplementary table S5, supplementary figs. S2 and S3, Supplementary Material online). Collectively, this points to a breeding history associated with relatively less intensive selection.

### Phylogenetic and Demographic Analyses

Principle component analysis (PCA) of ∼1.4 million unlinked SNP genotypes revealed that the Chinese native horses, Przewalski’s horses (PrZ) and Thoroughbred horses clustered in separate, equidistant groups (fig. 1*B*, upper panel). Restricting the analysis to Chinese native horses unveiled three major clusters (fig. 1*B*, main panel), with the first PCA axis separating the breeds of the Southwestern Chinese horses (SW, orange) from the others, and the second PCA axis separating the breeds from the QT Plateau (QT, blue) and those from the Northern China plains (NC, green). Horses from the Qinghai Province occupied an intermediate position along the second axis and appeared to be more mixed with the Tibetan horses than with the NC horses along the third PCA axis (supplementary fig. S4, Supplementary Material online). The Admixture analysis (Alexander et al. 2009) was largely consistent with the PCA results (supplementary fig. S5, Supplementary Material online) and supported both the similar genetic makeup of the Tibetan and Qinghai horses, as well as the presence of three major clusters, consisting of the NC, SW, and QT Chinese native horses.

We next investigated the phylogenetic relationships amongst the clusters identified within Chinese native horses. Both the ML-TreeMix tree (Pickrell and Pritchard 2012) and the distance-based Neighbor-Joining tree (Saitou and Nei 1987) were in line with the results of the PCA and Admixture analyses and supported a three-clade population structure, separating the southwestern montane horses, northern lowland horses and Qinghai/Tibetan horses (fig. 1*C* and *D*). The reliability of the Neighbor-Joining tree was estimated by 100 bootstrap pseudoreplicates. BEAST (Drummond and Rambaut 2007) phylogenetic inference based on the 4-fold degenerate (synonymous) nucleotide sites also supported the genetic structure around three main geographic areas of NC, SW and QT (supplementary fig. S6, Supplementary Material online). Interestingly, important migration weights that showed the magnitude of migration events, were inferred by TreeMix between breeds from the QT Plateau and the southwestern montane area (fig. 1*C*, supplementary fig. S7, Supplementary Material online). Whether this reflects the influence of the so-called “Ancient Tea-Horse Road,” which connected Tibet and Yunnan Province over the last thousand years (Yang 2004), remains to be tested. Yunnan, situated in Southwest China and bordering Tibet in the north, is famous for its Pu-erh tea. Interestingly, none of the six migration edges considered in TreeMix involved Przewalski’s horses (fig. 1*C*, supplementary fig. S7, Supplementary Material online), ruling out their significant (if any) genetic influence on Chinese horses. This is in line with ancient genome evidence showing that Przewalski’s horses represent the feral descent of Eneolithic horses that did not contribute to the genetic makeup of modern domestic horses (Gaunitz et al. 2018).

We next investigated the demographic history underlying the evolutionary origins of the three main population clusters identified. To achieve this, we compared three possible population scenarios showing each one of the three main clusters branching out first, and a fourth scenario in which all three clusters differentiated at the same time. The latter scenario was best supported by the ∂a∂i algorithm (Gutenkunst et al. 2009) (log-likelihood = –354,057 vs. <–390,000; supplementary table S6, Supplementary Material online) and fastsimcoal2 (Excoffier and Foll 2011) simulations (–3,677,890 vs. <–4,659,000; supplementary table S7, Supplementary Material online).

The four scenarios tested above considered that the three main lineages of Chinese native horses evolved in isolation after divergence. To accommodate the possibility of gene flow suggested by the TreeMix analyses, we further refined the fourth scenario, considering asymmetric migrations. This further improved the likelihood of the model (fig. 1*E* and supplementary fig. S8, Supplementary Material online) and indicated that the three lineages of NC, SW and QT horses diverged ∼3,700 years ago (average = 3,676 years BP, bootstrap 95% CI = 3,672–3,712 years ago; supplementary table S8, Supplementary Material online). This divergence time is consistent with 1) current time estimates for the expansion of the ancestors to all modern domesticated horses (Gaunitz et al. 2018), 2) the first archaeological evidence of draught horses in China during the early Bronze Age (∼4,300 BP to ∼3,800 BP) and of horse carriage in late Shang dynasty (∼3,300 BP to ∼3,000 BP, supplementary table S9, Supplementary Material online), 3) archaeological findings suggesting the presence of horses in Tibet in the second millennium BCE (Chen et al. 2015; Hu et al. 2019), and 4) the emergence of pastoral nomads in northwestern China (Chen et al. 2015). The best-supported demographic model also indicated asymmetric gene flow among the three clusters, with the largest migration contributions from QT and NC into SW, respectively (log-likelihood = –351,986, migration = 22.03 and 8.33 individuals/year, respectively, fig. 1*E*). This is consistent with the migration edges inferred by TreeMix. F3-statistics showed the gene flow between NC and SW and also between NC and QT. Furthermore, *D*-statistics supported the significant gene flow between SW and Tibetan (D [PrZ, SW; NC, Tibetan] = 0.0073, *Z* = 4.54), as well as between NC and QT (D [PrZ, NC; QT, SW] = −0.0031, *Z* = –2.81) (supplementary tables S10 and S11, Supplementary Material online).

### Genome-Wide Selection Scans for Adaptation to Life at High Altitude

**Fig. 2.**
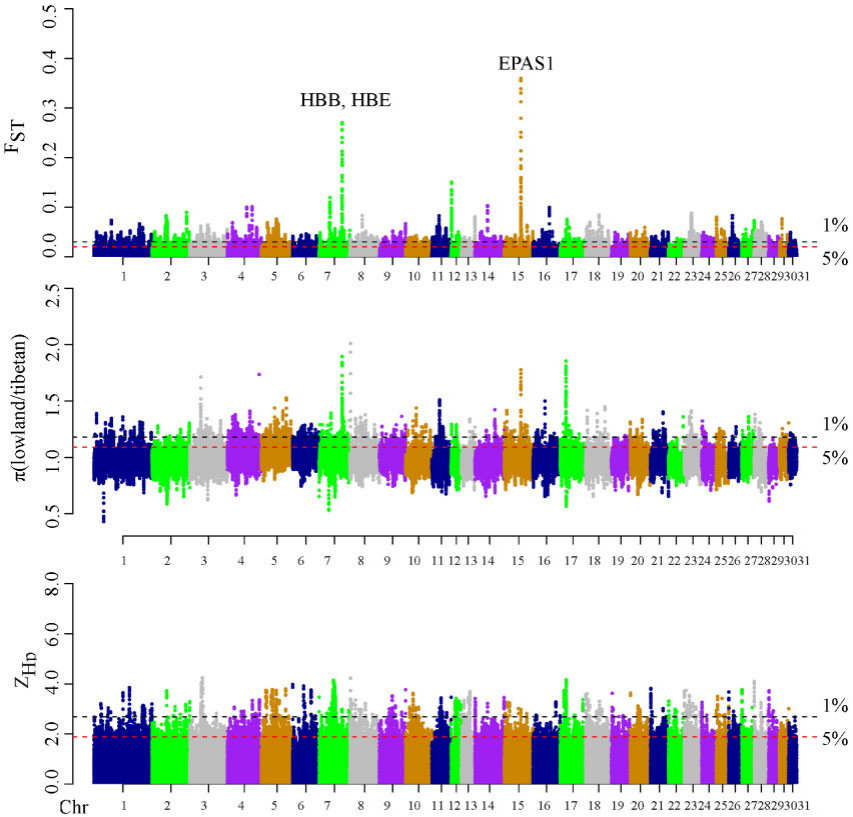
Positive selection scans for adaptation to high altitude hypoxia. Horses living at high altitude (QT) are compared with lowland controls (NC and SW). The population genetic differentiation *F*_ST_ values (*A*), the nucleotide diversity *θ*_π_ ratios (*θ*_π-LL_/*θ*_π-QT_) (*B*) and the transformed heterozygosity score ZH_P_ (*C*) are calculated within 100 kb sliding windows (step size = 15 kb). The significance threshold of selection signature was arbitrarily set to top 5% percentile outliers for each individual test and is indicated with red horizontal dashed lines. The black horizontal dashed lines display the top 1% quantile.

We next scanned the Tibetan horse genomes for signatures of positive selection, leveraging allele frequency differences and patterns of molecular diversity within 100 kb sliding windows (heterozygosity and nucleotide diversity) relative to lowland horse breeds (fig. 2). The top-5% selection candidates that were common to all three tests carried out represented a total of 159 windows spanning 440 protein-coding genes (supplementary tables S12–S15, Supplementary Material online). Enrichment analyses for Gene Ontology terms revealed that the hemoglobin complex (GO: 0005833), the oxygen transport (GO: 0015671), and the oxygen transport activity (GO: 0005344) categories were significantly overrepresented (*P*-values = 0.02–0.05, supplementary table S16, Supplementary Material online). This is consistent with the extremely hypoxic environmental conditions in the QT Plateau. Importantly, the genomic window containing the *EPAS1 (HIF2a)* locus was the top selection candidate identified (chromosome 15, fig. 2) whereas the second candidate region included the *HBE1* gene (chromosome 7, fig. 2). Both genes are known to be involved in oxygen transport and hypoxia adaptation (Wang et al. 2014).

### Annotation of Variants under Positive Selection in *EPAS1*

**Fig. 3.**
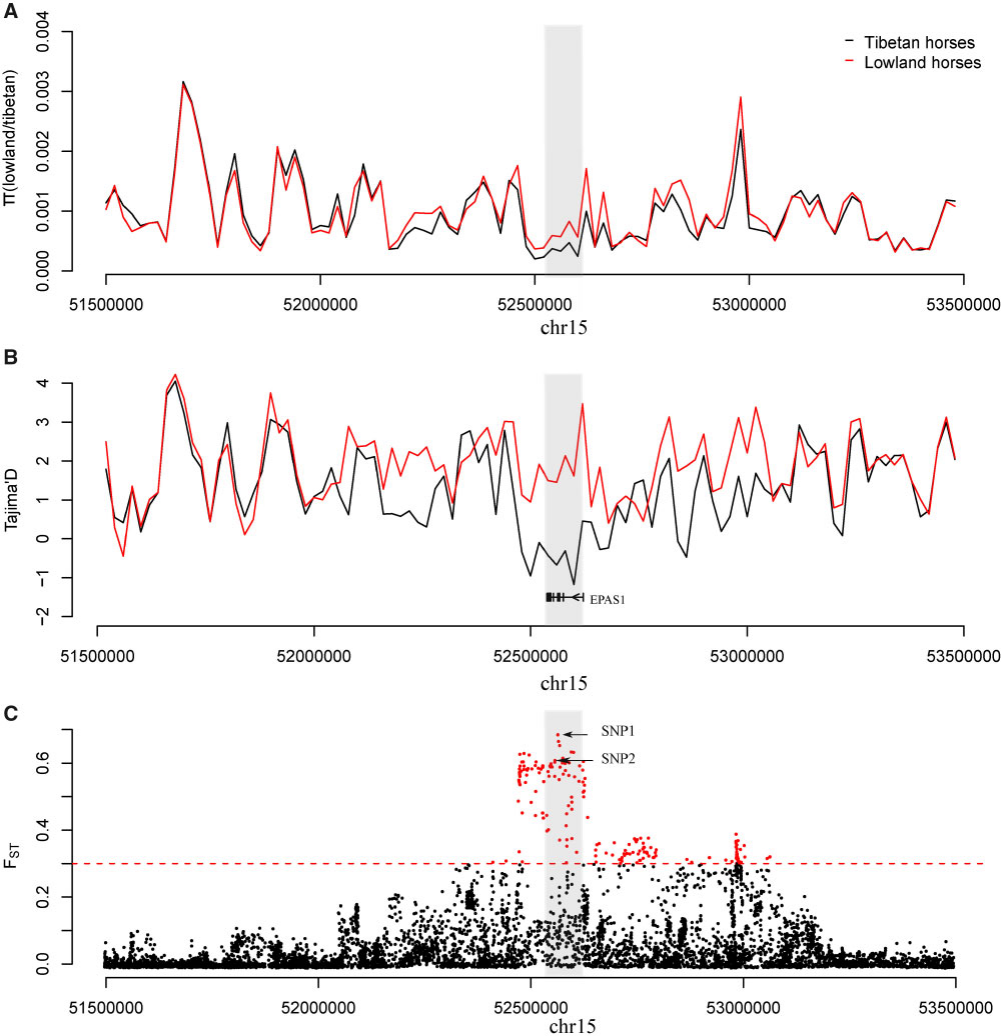
The strongest positive selection signatures around the *EPAS1* peak. The *θ*_π_ ratio (*θ*_π-LL_/*θ*_π-QT_) (*A*), Tajima’s *D* (*B*) and *F*_ST_ value (*C*) are plotted against the peak position from 51.5 Mb to 53.5 Mb on chromosome 15. Both π ratio and Tajima’s *D* values were based on a 20 kb window and a 20 kb step. The black and the red lines represent the Tajima’s *D* values for high-altitude and lowland horses, respectively. The gray columns represent the strongest positive selection signatures in the region considered. The small black boxes and short lines represent the gene structure of *EPAS1*, which is the only gene within the strongest selective signal. The red dot represents the significant threshold of *F*_ST_ value per SNP > 0.3 and the red dashed line represents the *F*_ST_ threshold. The top two SNPs are noted by black arrows.

**Fig. 4.**
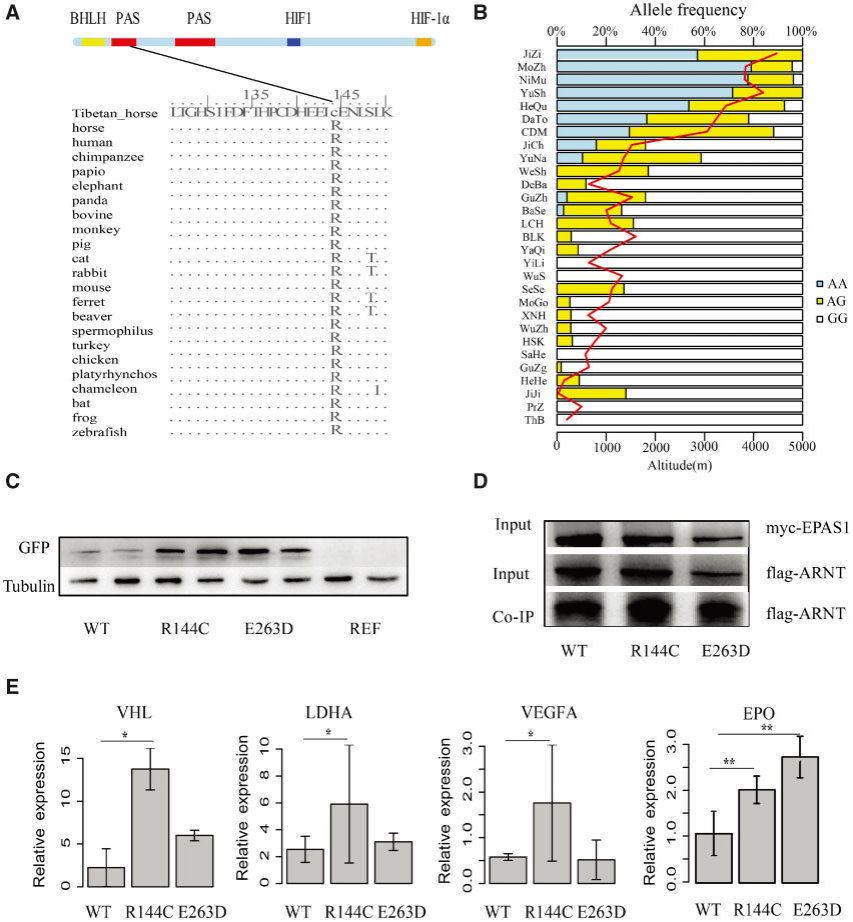
Annotation and validation of the *EPAS1* missense SNPs showing positive selection signatures. (*A*) EPAS1 protein sequence analysis. The protein coordinates are based on the ENSECAT00000015683.1Ensembl protein. The upper panel shows the Pfam domains of the EPAS1 protein, including the Per-Arnt-Sim (PAS) domains (red), the basic helix-loop-helix (HLH) domain (yellow), the hypoxia-inducible (HIF) domain (blue) and the C-terminal transactivation (CTAD) domain (orange). Protein sequence polymorphisms present in Tibetan horses and 22 vertebrates are provided. (*B*) Genotypes were determined using the KASP technology in Przewalski’s horses, Thoroughbred horses and 27 Chinese native horse breeds (*N* = 908 horses). The region and altitude information are indicated by the red line. (*C*) Western-blotting analysis of the A549 cell lysates transfected with the GFP-tagged recombinant plasmid of WT, R144C and E263D or the empty vector. WT, R144C and E263D represent the plasmid overexpressing the wild-type EPAS1 protein, SNP1 mutant and SNP2 mutant protein, respectively, REF represents the empty vector. The antiGFP and antiTubulin antibodies were used to measure the protein expression of EPAS1 and the internal reference protein Tubulin, respectively. (*D*) Validation of EPAS1 and ARNT interactions. A549 cells were cotransfected with myc-tagged EPAS1 and flag-tagged ARNT for 48 h, followed by immunoprecipitation against myc tag and immunoblotting against flag and myc. (*E*) The qPCR gene expression of the *EPAS1* downstream genes, including *VEGFA, VHL* and *LDHA* in the transfected A549 cells. *EPO* expression levels were measured in HepG2 cells. * and ** displayed the statistical significances of *P-values *<* *0.05 and 0.01, respectively.

We next sought to further refine the selection targets within the two top-candidate regions (*EPAS1* and *HBE1*), possibly at the individual SNP level by using three different methods, *θ*_π_ ratio of the low-altitude horse breeds to the high-altitude horse breeds (*θ*_πLL_/*θ*_πQT_), Tajima’s *D* and *F*_ST_. We noticed that two SNPs within the most significant selection region represent missense variants of the *EPAS1* gene (fig. 3 and supplementary tables S21–S23, Supplementary Material online). They are located at positions 52,566,293 and 52,552,723 of chromosome 15, within two extremely conserved PAS domains, and segregate within two different haplotypes (fig. 4*A*, supplementary figs. S9 and S10, Supplementary Material online). The EPAS1 protein is highly conserved among mammals, with two heterodimerization PAS domains showing close to 100% sequence similarity amongst 22 different species, including humans and horses living at low altitude. The missense variants (hereafter referred to as SNP1 and SNP2, respectively) are not present in Przewalski’s horses, suggesting that they arose in an independent genetic background.

To further investigate the possible functional consequences of SNP1 and SNP2 variants, we investigated whether they showed any association with high altitude, extending our analysis to a more comprehensive panel of 908 horses originating from 29 populations (min. 20 animals per population, supplementary table S17, Supplementary Material online). This analysis supported a strong correlation between the frequencies of both missense variants and altitude (*P*-value *=* 6.02E–05 and 2.00E–07, respectively). Tibetan horse breeds living >4,000 m showed the highest allelic frequencies of both variants (almost 0.8) whereas horses living at low-altitude (below 1,000 m) showed the lowest (≤0.05). Allelic frequencies decreased to 0.6–0.7 in the QT breeds (3,000–3,500 m) and to 0.2–0.3 in the Yunnan breeds (1,500 m) associated with the Tea-Horse Route (fig. 4*B* and supplementary fig. S9, Supplementary Material online).

Using the same approach, we also explored the possible association of one missense SNP present in the *HBE1* gene (SNP3). While the association was significant, it was not as strong as those detected at the SNP1 and SNP2 of *EPAS1* gene (*P*-value *=* 5.5E–04) (supplementary figs. S11 and S12, Supplementary Material online). This suggests that the two variants of *EPAS1* likely have larger biological effects than *HBE1*. The PolyPhen-2 score of SNP1 (R144C) is larger than that of SNP2 (E263D) (supplementary fig. S13, Supplementary Material online), suggesting larger functional effect for the former. It may thus represent the major genetic mutation underlying high-altitude adaptation in Tibetan horses.

### Functional Effects of the Two Selected *EPAS1* Missense Mutations

We next sought to functionally assess the effects of the R144C and E263D missense mutations in adenocarcinoma human alveolar basal epithelial cells (A549). This cell line was selected as it shows high expression levels of the EPAS1 protein and its target genes (Sato et al. 2002). Western blot analyses revealed that the expression levels of the two GFP tagged-mutant EPAS1 proteins carrying each individual variant were higher than wild-type (fig. 4*C* and supplementary fig. S14, Supplementary Material online). This suggests that both mutations can stabilize the EPAS1 protein by altering the structure of PAS domains.

Under hypoxic conditions, the EPAS1 protein is known to be translocated into the nucleus, where it binds the aryl hydrocarbon receptor nuclear translocator *ARNT* (*HIF-1β*) via PAS domains (Luo and Shibuya 2001). The resulting heterodimer can then bind to DNA hypoxia response elements (HRE) where it initiates the transcription of key genes involved in the response to hypoxia, such as *erythropoietin (EPO)*, *lactate dehydrogenase A (LDHA)*, *endothelin (EDN1)*, *vascular endothelial growth factor A (VEGFA)*, *von Hippel-Lindau (VHL)* and *prolyl pydroxylase domain-containing protein 2 (PHD2*, also *EGLN1*; Lee et al. 2011). We thus hypothesized that the two mutants identified may influence the structure of the PAS domains and the heterodimerization affinity of EPAS1 for ARNT.

To test this hypothesis, we co-expressed myc-tagged EPAS1 and flag-tagged ARNT proteins in A549 cells and carried out a coimmunoprecipitation assay using antimyc antibody. This was aimed to isolate the heterodimer formed and assess whether any of the two mutations could affect the interaction (fig. 4*D*). Coimmunoprecipitation assays confirmed the strong protein–protein interaction between the wild-type EPAS1 and ARNT proteins. This interaction was greatly enhanced in the *EPAS1* R144C mutant but did not significantly change for the E263D mutant. This suggests that the R144C missense mutation, but not the E263D missense mutation, increases the heterodimerization affinity of EPAS1 for ARNT, in line with the PolyPhen-2 scores of the variants.

We further examined whether the *EPAS1*missense mutations could alter the transcriptional response to hypoxia using RT-qPCR assays (fig. 4*E*). The mRNA expression levels of four out of the seven downstream genes tested (*EDN1, LDHA*, *VHL* and *EPO*) were significantly increased in R144C transfected cells (fig. 4*E*). No significant changes in RNA expression levels were observed in the E263D transfected cells or in the three other genes tested (*VEGFA, EGNL2* and *EGNL3*). This confirmed that the R144C missense mutation as a gain-of-function mutation, which amplifies the transcriptional activity mediated by *EPAS1* in response to hypoxia.

### *EPAS1* Missense Mutations Correlate with Improved Oxygen Carrying Traits and High Anaerobic Metabolic Capacity in Tibetan Horses

**Fig. 5.**
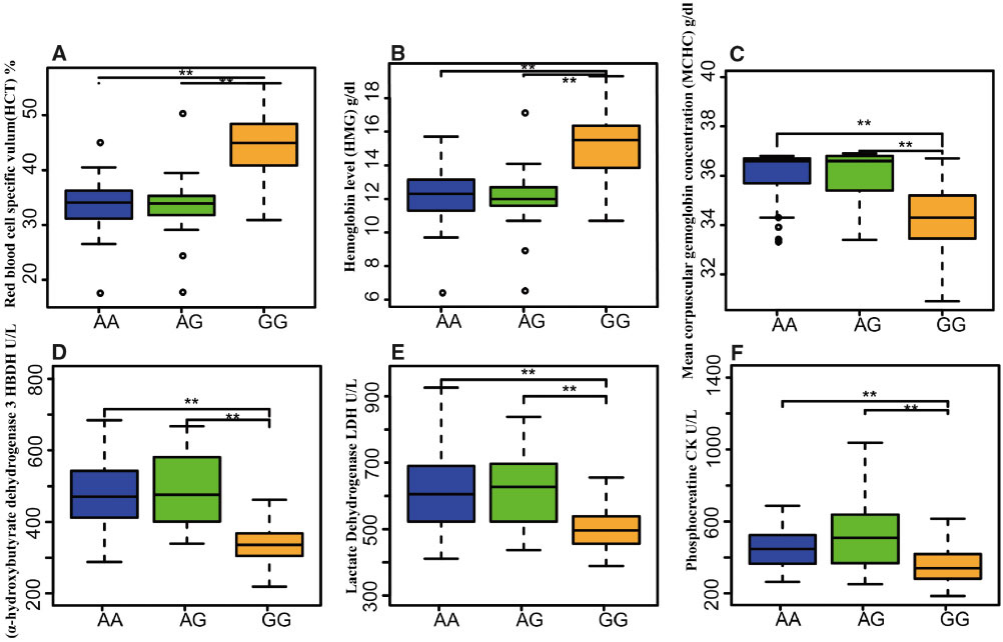
Genotype–phenotype association of the EPAS1-R144C mutation. Association analysis of the EPAS1-R144C SNP (SNP1: mutant allele, A; reference allele, G) with the individual hemoglobin level and blood physiological values in Hequ (HeQu) and Guanzhong horses (GZg). We found a significant association between the genotypes and HCT (*A*), HMG (*B*), MCHC (*C*), HBDH (*D*), LDH (*E*) and CK (*F*). ** Displays the statistical significance of *P-value *<* *0.01 according to the ANOVA *F*-test.

We next measured a total of 23 physiological blood characteristics in the Tibetan (*N* = 88) and lowland horses (*N* = 85) that were also genotyped at SNP1 (the G allele results in the presence of an arginine, R at amino acid position 144 whereas the A allele introduces a cysteine, C at that position). We found a significant statistical association between the *EPAS1* SNP1 (R144C) mutation and hemoglobin levels (HMG, *P*-value = 1.11E–17), the volume of red blood cells (RBCV, *P*-value = 1.20E–21), the mean corpuscular hemoglobin concentration (MCHC, *P*-value = 5.12E–13) and three other hematological parameters (HBDH, *P-value* = 4.79E–16; LDH, *P-value* = 2.80E–9; CK, *P-value* = 6.33E–5). Interestingly, the different hematological parameters investigated were found to show different associations with particular *EPAS1* genotypes, with A homozygous carriers showing lower RBCV and HMG but larger MCHC (and α-HBDH3, LDH and CK levels; fig. 5, supplementary tables S18 and S19, Supplementary Material online). Additionally, Tibetan horses showed ∼15% higher CK levels than lowland horses, possibly reflecting the absence of oxidative stress observed in response to hypoxia (fig. 5 and supplementary table S19, Supplementary Material online). Finally, the mutation of R144C in *EPAS1* appears to account for 25.45% of the hematocrit (HCT) variance, 12.71% of the HMG variance and 14.72% of the MCHC variance (supplementary table S24, Supplementary Material online).

## Discussion

In this study, we sequenced the genomes of 138 horses from 17 Chinese breeds spanning a wide range of altitudes, from the lowlands of the NC plains to the QT Plateau. Our extensive genome data set allowed us to identify a total of ∼10.3 million SNP variants (plus ∼2.3 M indels and CNVs) that helped unveiling the population structure of Chinese horses at unprecedented levels. In line with previous work based on 27 microsatellites (Ling et al. 2011), we found that the genetic landscape matched broad geographic subdivisions, with three main clusters in the NC, SW and the QT regions (fig. 1*A*–*E*). Statistical modeling revealed that the three main clusters split simultaneously ∼3,700 years ago. This follows in ∼500 years the early demographic expansion of the lineage ancestral to all modern domestic horses (Sanchez-Mazas 2012) and predates the earliest archaeological evidence of horse chariotry in the early Shang dynasty by ∼500 years (Sanchez-Mazas 2012). Altogether, this indicates that domestic horses radiated throughout the southern, northern and montane areas of China soon after their first arrival in the country. Determining whether domestic horses first arrived to China harnessed to spoke-wheel chariots or mounted purpose will require further archaeological work.

Scanning the genome of high-altitude Tibetan horse breeds for signatures of positive selection revealed *EPAS1* and *HBE1* genes within the top-candidates (fig. 2). The same selection targets have been described in a number of other organisms, including Sherpa humans (Horscroft et al. 2017), Tibetan dogs (Wang et al. 2014) and yak (Qiu et al. 2012). This repeated selection of similar physiological and metabolic pathways in response to high-altitude hypoxia provides a textbook example of parallel evolution (Beall et al. 2010; Wang et al. 2014; Zhang et al. 2014). Previous studies have established a key role for *EPAS1* in mediating the blood physiology of Tibetan Sherpas (Horscroft et al. 2017). *EPAS1* SNP variants unique to native Tibetans as well as two gain-of-function mutations in the *EGLN1/PHD2* negative regulator of HIF, which include *EPAS1* (Lorenzo et al. 2014), have indeed been found to be significantly associated with lower HMGs (Beall et al. 2002). Similar to human studies on large Tibetan cohorts (Yang et al. 2017), we found that both HMG and HCT of Tibetan horses were lower than those of lowland horses (fig. 5*B* and *C*). In contrast to humans, the MCHC of Tibetan horses was, however, higher, a pattern reminiscent of what is also observed in Tibetan dogs (Gou et al. 2014), Tibetan sheep (Wei et al. 2016) and Tibetan Cashmere goats (Song et al. 2016). This reduced number of red blood cells (RBCs) found in the Tibetan horses likely facilitates blood circulation, while the increased HMG per cell ensures that more oxygen can be carried, delivered and ultimately consumed. The increased LDL and α-HBDH3 levels measured in Tibetan horses suggest indeed greater capacity for anaerobic glucose fermentation and ATP production relative to lowland horses, similar to what is observed in human Sherpas (Horscroft et al. 2017).

At the molecular level, our work reveals that two nonsynonymous mutations located in the otherwise conserved PAS-A and PAS-B domains of the *EPAS1* gene are key adaptations to hypoxia in Tibetan horses. These mutations are associated with higher stability and/or activity of the EPAS1 protein in transfected A549 cells, in line with previous observations (Prabhakar and Semenza 2012). This ultimately results in increased transcription of key downstream gene targets within the HIF signaling pathway involved in the response to hypoxia, such as *VEGFA, LDHA* and *VHL* (Bigham and Lee 2014). Interestingly, the SNP variants underlying hypoxia adaptation in Tibetan horses were found to segregate at low but nonzero frequencies in a number of lowland breeds. These mutations may have different effects on the regulation of erythropoiesis as well as on metabolism, which is another major target of HIF regulation. Future genomic selection programs in commercial breeds carrying these variants might help overcome the physiological deficiency of lowland animals when transported to high altitude.

## Conclusions

Our study provides the first in-depth whole-genome sequence analysis of Chinese native horse breeds. It includes a total of 138 individual genomes spread across 17 breeds and spans the entire altitudinal gradient, from the lowland region of northeast China to the high-altitude QT Plateau. Patterns of neutral molecular diversity reveal that China was populated by the ancestral lineage of modern domestic horses in the early Bronze Age, ∼3,700 years ago, and that an early radiation in the southern, northern and montane QT range formed the three main clusters of present-day diversity. Genomic regions showing signatures of positive selection in QT horses revealed *EPAS1* as the top candidate for adaptation to life at high altitude. Genotyping data across a large panel of 908 lowland and high-altitude horses revealed that two missense variants in two PAS domains of the *EPAS1* gene are strongly associated with altitude, and reach close-to-fixation (∼80%) frequencies in high-altitude Tibetan horses. Functional assays demonstrate that these variants result in higher stability and heterodimerization activity of the EPAS1 protein, as well as low RBCVs, high HMGs and increased anaerobic metabolism capacities. Our approach showcases the power of population genomic tools and selection scans over more classical genome-wide association studies to shortlist a number of selection candidates that can be further functionally validated in a cost-effective manner. Our study adds the horse to the list of other Tibetan mammals showing *EPAS1* as a key adaptive driver to life at high altitude. This not only provides a textbook example of convergent evolution but also offers new opportunities to the breeding industry in the form of novel selection targets for improving lowland horse welfare following exposure to hypoxic environments.

## Materials and Methods

### Ethics Statement

Handling and sampling of horses were carried out in full respect to animal welfare. All procedures involving the handling of horses were approved by the Animal Care and Use Committee of the Chinese Academy of Agricultural Sciences and the Ministry of Agriculture of the People’s Republic of China (IASCAAS-AE-03).

#### Samples

A total of 138 horses representing 126 Chinese native horses, five Przewalski’s horses and seven Thoroughbred horses were selected for whole-genome sequencing (supplementary table S1, Supplementary Material online and fig. 1*A*). Half of the 126 Chinese native horses live above 3,300 m in the QT Plateau, with 35 animals from Tibet (10 Jiangzi, JiZi; 10 Langkazi, LKZ; 6 Mozhu, MoZh; and 9 NiMu, NiMu) and 26 from Qinghai (6 HeQu; 10 Datong, DaTo; and 10 Chaidamu, CDM). All other native horses investigated originated from low-altitude areas (below 1,000 m), mainly from the diverse geographic areas of China, including NC, with 17 horses from Xinjiang (YiLi and YaQi), 7 from Jilin (ELC), 8 from Inner Mongolia (MoGo) and 7 from Mongolia (WMG); and SW China, with 8 horses from Sichuan (JiCh), 8 from Shaanxi (NiQi) and 10 from Guangxi (DeBa). A minimum of two separate flocks were sampled for each breed or location, and parent/offspring pairs were excluded. Closely related individuals were also disregarded based on available microsatellite data (Ling et al. 2011). This large collection of horse samples was further used in the current study to validate the identified variants (supplementary table S17, Supplementary Material online).

#### Whole Genome Resequencing Analysis

The extracted DNA from each collected sample was sequenced on Illumina HiSeq XTen/2500 instruments at BerryGenomics Company (Beijing, China). High-quality trimmed read pairs were aligned against the reference horse genome assembly EquCab 2.0 genome (Wade et al. 2009) using BWA (version: 0.7.12) with default parameters (Li et al. 2009). Only read pairs that showed mapping qualities superior to 20 and that were identified as uniquely aligned by the Picard Mark Duplicates (picard.sourceforge.net, version 1.86) were further considered for calling genotypes. This was carried out using two methods, including the Genome Analysis Toolkit version 2.4 (McCormick et al. 2015) and SAMtools (Zhou et al. 2015), and the set of genotypes common to both methods was retained to minimize the false positive SNP calls. Specifically, SNPs were retained if 1) their confidence scores (QD) was superior or equal to 20; 2) their Phred-scaled *P*-values of the Fisher’s exact test aimed at detecting strand bias (FS) was inferior or equal to 10; 3) the *Z*-scores of the Wilcoxon rank sum test of Alt versus Ref read position bias was superior or equal to –8; 4) the SNP quality scores of each individual SNP were greater than average; and 5) they were biallelic and found at a minimal allele frequency of 5%. The detailed assembly for SNP, indel and CNV calling are described in the supplementary methods, Supplementary Material online.

#### Population Structure and Phylogenetic Analysis

All SNPs were pruned using PLINK (Version: 1.90 b), considering window sizes of 1,000 variants, a step size of 5 and a pairwise *r*^2^ threshold of 0.5 (--indep-pairwise 1000 5 0.5). This retained a total of 1,449,645 independent SNPs for all subsequent analyses. Principal component analysis (PCA) was conducted using the GCTA 1.91 software (Xu et al. 2011). For the first PCA plot, all 138 animals were used, with the first three principal components cumulatively explaining 11.95% of the total variance. Population structure was evaluated using ADMIXTURE (Version: 1.3.0; Alexander et al. 2009), considering a total of 10,000 iterations and two to nine genetic clusters (*K*). The Neighbor-Joining tree was constructed using PHYLIP 3.68 (evolution.genetics.washington.edu/phylip.html). MEGA5 (Tamura et al. 2011) and FigTree software (tree.bio.ed.ac.uk/software/figtree/) were used to visualize the phylogenetic trees. BEAST v1.10 (Drummond and Rambaut 2007) was run on the data set using a log-normal relaxed clock model, for 100,000,000 states to generate the maximum clade credibility (MCC) tree (sampling frequency = 1/1,000, burnin = 10%). We used the HEK_GAMMA model of nucleotide substitution, with four rate categories for gamma-distributed rates across sites. The MCC tree returned from BEAST was then visualized using SpreaD3 (Bielejec et al. 2011). SNPs at 4-fold degenerate synonymous sites were used in the BEAST analyses in order to reduce the data size and make them computationally manageable instead of SNPs in noncoding regions used in ∂a∂i. For further information, see the supplementary methods, Supplementary Material online.

#### Demographic History Inference Using ∂a∂i

We reconstructed the Chinese native horse demographic history using the diffusion approximation method for the allele frequency spectrum (SFS) implemented in ∂a∂i. This program can also assess the statistical support of isolation models versus models including migration. ∂a∂i analyses were restricted to SNPs located in noncoding regions to mitigate potential effects of selection that could interfere with demographic inference (Zhang et al. 2018). We considered the three major phylogenetic clusters, consisting of NC, SW and QT, and tested four possible demographic scenarios, each depicting different divergence orders of the three horse clusters. We selected the model showing the highest log-likelihood value as the optimal model (Model 4). We also used fastsimicoal2 (Excoffier and Foll 2011) to confirm our model selection. The detailed models, statistics and python scripts to implement the simulations are shown in supplementary tables S7, S8 and supplementary methods, Supplementary Material online. After model selection, scaled parameters for the best-supported model were transformed into the real values using the same *μ* = 7.242e–9 (per site per generation) and *g* = 8 (year) as described in the study (Orlando et al. 2013). Gene flow was modeled as discrete migration events at a certain time after population divergence.

#### Migration Events

Admixture analysis was conducted by TreeMix (Version: 1.12; Pickrell and Pritchard 2012) at the population level. After converting the PLINK SNP matrix to TreeMix-format by the plink2treemix.py python script, we constructed the ML tree using Przewalski’s horses as an outgroup, both in the absence (*m* = 0) and the presence (1 ≤ *m* ≤ 6) of migration.

#### Genomic Selection Signatures

We scanned the Tibetan horse genomes for signatures of positive selection using the population-differentiation value (*F*_ST_; Weir and Cockerham 1984), the nucleotide diversity (*θ*_π_) ratio (*θ*_π__-LL_/*θ*_π__-QT_) (Nei and Li 1979) of the lowland (LL) group to QT and the transformed heterozygosity score (ZH_P_). The window-based ZH_P_ approach was calculated as follows: ZH_P_ = (Hp – *μ*Hp)/σHp, where *μ* is the overall average heterozygosity and *σ* is the standard deviation of all windows within each group (Rubin et al. 2012; supplementary tables S12–S14 and supplementary methods, Supplementary Material online). Evidence for positive selection in response to high-altitude hypoxia was evaluated by contrasting differentiation indices between the Chinese native horses from the QT Plateau (>3,300 m) and the LL Chinese horses (<1,000 m). All diversity indices (*F*_ST_, ZH_P_, and *θ*_π_) were calculated using 100 kb sliding window (step size = 15 kb) using VCFTools (–fst-window-size, –fst-window-step, and –weir-fst-pop; Danecek et al. 2011; Cavadas et al. 2019). The significance threshold was set to the top 5% for each individual test. Only those windows that were returned positive in all three tests were further considered as potential selection candidates, and the corresponding genes were used in Gene Ontology enrichment analysis (supplementary table S15 and supplementary methods, Supplementary Material online).

#### Functional SNPs

*θ*_π_ ratio (*θ*_π__-LL_/*θ*_π__-QT_), *F*_ST_ and Tajima’s *D* values were calculated using VCFTools for each SNP position within the top candidate regions under selection in Tibetan horses. To predict functional candidates, those variants returning the most significant signatures were classified according to their evolutionary conservation scores among 22 mammals. The PolyPhen value was calculated by the database (genetics.bwh.harvard.edu/pph2/) to assess the possible functional consequences of each nonsynonymous variant.

#### Validation in the Extended Populations

Three specific mutations within the *EPAS1* gene (R144C, SNP1; E263D, SNP2) and the *HBE1* gene (V147A, SNP3), respectively, were successfully genotyped in the extended populations consisting of 908 horses by the Kompetitive Allele Specific PCR (KASP) genotyping platforms at Beijing Compass Biotechnology Co. Ltd. (Beijing, China; Semagn et al. 2014). All the samples tested are described in supplementary table S17 and supplementary methods, Supplementary Material online.

#### Physiological Association

We collected two sets of jugular venous blood samples from 88 adult Hequ horses (HeQu) living at an altitude of 3,500 m and 85 adult Guanzhong horses (GZg) living at an altitude below 100 m. The first set was collected in coagulant tubes, whereas the other was collected in anticoagulation tubes (BD, 3678124). We used the IDEXX Vet Autoread Blood Analyzer (IDEXX, America) on-site to measure six hematological parameters on the former set. These included RBC counts, HMG, HCT, white blood cell (WBC) and MCHC. All on-site measurements were completed within 2 h after blood collection. The other functional assays were conducted in the laboratory on the second blood set, following serum preparation within 12 h after blood collection. The serum was stored at –20 °C until further blood biochemical parameters were measured using a HITACHI 7080 Autoread Biochemical Analyzer (HITACHI, Japan) following the manufacturer’s protocol. The blood parameters measured included glutamic-pyruvic transaminase (ALT), glutamic oxaloacetic transaminase (AST), total protein (TP), A/G, alkaline phosphatase (ALP), serum lactate dehydrogenase (LDH), creatine kinase (CK), α-hydroxybutyrate dehydrogenase (HBDH), blood glucose (CLU), total cholesterol (CHOL), triacyl glycerol (TG) and calcium (Ca). Additionally, all samples were further genotyped for *EPAS1* R144C (SNP1), E263D (SNP2) and *HBE1*V76A (SNP3) to test for possible genetic association with any of the physiological parameters measured.

#### Statistical Analysis

The statistical test was performed by using ANOVA methods (R packages, stats). Further information pertaining to the blood tests conducted here is provided in supplementary tables S18 and S19, Supplementary Material online. The fixed and random model Circulating Probability Unification (FarmCPU; Liu et al. 2016) were used to calculate the effective values (effB). This approach handles confounding issues between covariates and test markers using both the fixed effect model (FEM) and a random effect model (REM). Sex correction was used as FEM in this study. Then, the effB in the FarmCPU result was regarded as the allele substitution effect, and the proportion of phenotypic variance explained by each significant SNP was estimated as follows:

$$\text{VAR}(\%)=\frac{2pq\beta^{2}}{S^{2}}\times 100$$

where *p* and *q* are the allele frequencies, *β* is the estimated allele substitution effect, and *S^2^* is the sample phenotypic variance (Velie et al. 2018).

#### Cell Maintenance

A549 cells were obtained from the Peking Union Medical College Hospital and were propagated in Roswell Park Memorial Institute 1640 (RPMI 1640) medium supplemented with 10% heat-inactivated fetal bovine serum and penicillin (0.2 U/ml)/streptomycin (0.2 μg/ml)/l-glutamine (0.2 μg/ml) (Gibco, USA).

#### Constructs and Mutagenesis

The pcDNA3.1 vector with a GFP flag was purchased from Invitrogen Company, whereas the myc-tagged and flag-tagged pcDNA3 vectors were provided by Dr Qinghe Li (Wang et al. 2016). The wild-type *EPAS1* gene was amplified from the cDNA of lowland humans. The mutagenesis of the *EPAS1* sequence (R144C and E263D) was achieved through site-directed mutagenesis (Genewiz) and verified by Sanger sequencing. Briefly, the complementary oligo deoxyribo-nucleotide primers were designed at the mutation sites, and then polymerase chain reaction (PCR) was used to generate fragments including the mutations. After double DNA digestion with NheI and BamHI (Beyotime), the EasyGeno Rapid Recombinant Clone Kit (VI201, TIANGEN) was used to subclone the wildtype *EPAS1* cDNA, the SNP1R144C sequence (G to A) or the SNP2 E263D sequence (T to A) into the pcDNA3.1 plasmid (Invitrogen) for the overexpression analysis. For the subsequent co-immunoprecipitation experiment, these three sequences of the *EPAS1* gene were subcloned into the myc-tagged plasmid, and the human *ARNT* gene was subcloned into the flag-tagged plasmid. All the recombinant plasmids were purified using the EndoFree Plasmid Mega or Giga Kits following the manufacturer’s instructions (Qiagen). Transfection, Western blotting and quantitative PCR analysis were performed according to the supplementary methods, Supplementary Material online. As the gene *Erythropoietin (EPO)* is only expressed in HepG2 cell, we also conducted transfection and RT-PCR in HepG2 cells to monitor *EPO* levels in the wild type, R144C and E263D EPAS1 backgrounds.

#### Co-Immunoprecipitation

Cells co-expressing myc-tagged EPAS1 mutants and flag-tagged ARNT proteins were lysed with RIPA buffer (50 mM Tris–HCl, pH 7.4, 150 mM NaCl, 0.25% deoxycholic acid, 1% NP-40, 1 mM EDTA, and 0.5% SDS supplemented with protease inhibitor) after two consecutive washing steps with PBS and centrifuged at 4 °C for 20 min (14,000 rpm). The supernatant was incubated under agitation at 4 °C for 2 h with 20 µl Protein A/G bead slurry (Abmart) that was prewashed with RIPA buffer 4 times. The beads were then centrifuged at 4 °C for 2 min to obtain a precleared supernatant. A fraction of the latter was frozen at –80 °C and served as the “Input” for Co-IP experiments, whereas the remainder was immunoprecipitated with 20 µl prewashed antimyc linked affinity gel beads (Abmart) at 4 °C for 2 h or overnight with rotation. After several washing steps (i.e., rinsing twice and washing twice for 30 min at 4 °C with rotation), the gel beads together with the linked EPAS1 complexes were denatured for WB analysis with an antiflag antibody and antimyc mouse monoclonal antibody (Abmart) to detect the protein interaction between the *EPAS1* mutants and *ARNT* (Wang et al. 2016).

## Data Availability

All the whole genome Illumina sequencing reads have been deposited in the Sequence Read Archive (https://www.ncbi.nlm.nih.gov/sra; last accessed July 8, 2019) with the accession codes (BioProject ID: PRJNA515160). The variation data reported in this paper have been deposited in the Genome Variation Map (GVM) in Big Data Center, Beijing Institute of Genomics (BIG), Chinese Academy of Science, under accession numbers GVM000026. Corresponding VCF files are publicly accessible at http://bigd.big.ac.cn/gvm/getProjectDetail? project=GVM000032; last accessed July 8, 2019. The FTP site (ftp://219.239.103.28/User name: ftpuser Password: ftpadmin) is available for downloading all the processed bam files.

## Supplementary Material

Supplementary data are available at *Molecular Biology and Evolution* online.

## Supplementary Material

msz158_Supplementary_Data
