# Supplementary material for: EPAS1 Gain-of-Function Mutation Contributes to High-Altitude Adaptation in Tibetan Horses
Source: Mol Biol Evol. 2019 Jul 4;36(11):2591–603. doi: 10.1093/molbev/msz158 (PMC6805228; doi:10.1093/molbev/msz158)
Supplement: msz158_Supplementary_Data [file msz158_supplementary_data.zip › Supplementary Information.docx]

Supplementary Information for

**EPAS1 gain-of-function mutation contributes to high-altitude adaptation in Tibetan horse**

Xuexue Liu^1,2#^, Yanli Zhang^1,2#^, Yefang Li^1,2#^, Jianfei Pan^1,2^, Dandan Wang^1,2^, Weihuang Chen^3^, Zhuqing Zheng^3^, Xiaohong He^1,2^, Qianjun Zhao^1,2^, Yabin Pu^1,2^, Weijun Guan^1,2^, Jianlin Han ^2,4^, Ludovic Orlando^5,6*^, Yuehui Ma^1,2*^, Lin Jiang ^1,2*^

^1^Institute of Animal Science, Chinese Academy of Agricultural Sciences (CAAS), Beijing 100193, P. R. China

^2^ CAAS-ILRI Joint Laboratory on Livestock and Forage Genetic Resources, Institute of Animal Science, Chinese Academy of Agricultural Sciences (CAAS), Beijing, 100193, P.R. China

^3^ College of Animal Science and Technology, Northwest A&F University, Yangling, Shaanxi 712100, China

^4^International Livestock Research Institute (ILRI), Nairobi, 00100, Kenya

^5^Centre for GeoGenetics, Natural History Museum of Denmark, University of Copenhagen, Copenhagen, Denmark.

^6^Laboratoire AMIS, CNRS, UMR 5288, Université Paul Sabatier (UPS), Toulouse, France.

**Running title:** Genetic adaptation to high-altitude in Tibetan horse

# These authors contribute equally to this article.

*Co-corresponding author:

E-mail: jianglin@caas.cn (Lin Jiang)

E-mail: yuehui.ma@263.net (Yuehui Ma)

E-mail: ludovic.orlando@univ-tlse3.fr (Ludovic Orlando)

**This PDF file includes:**

Methods

Figures. S1 to S14

**Methods**

**Sample information.** In this study, we sequenced a total of 138 horses representing 126 Chinese native horses, five wild Przwalski’s horses (PrZ), and seven Thoroughbred horses (ThB) were recruited for our study.About 50 percent of these reside in Qinghai-Tibet plateau above 3,300 m, with 10 horses originated in Jiangzi of Tibet (JiZi) with average altitude is 4,472 m, 10 in Langkazi (LKZ) of 4,000 m, 6 in Mozhu (MoZH) of 3,835 m, 9 in Nimu of Tibet (NiMu) of 3,818 m, 6 in Hequ (HeQu) from Qinghai province of 3431m, 10 in Datong (DaTo) from Qinghai of 3,243 m and 10 in Chaidamu (CDM) from Qinghai of 3048m. The other 50 percentage of native horses reside in the diverse geographic areas of China, below 1,000 m, including 17 horses originated in Xinjiang (YiLi and YaQi) with average altitude of 646m and 1,089 m, 8 from Inner Mongolia (MoGo) with altitude of 1,057 m, 7 in Mongolia (WMG), 7 in Jilin (ELC) with altitude of 482 m, 26 from Sichuan (JiCh) (1,523 m), Shaanxi (NiQi) (811 m) and Guangxi (DB) (646 m).

**Resequencing and construct library**

Total of 138 individuals were included in our study, among these individuals, 10 of them (NQ9916, DB35, JC5, PrZ6, ThB16, WMG8, ELC21, WZ6, YL2, YQ29) were sequenced with a high depth of ~20X to facilitate SNP calling. Tibetan horses including CDM (10), HeQu (6), JiZi (6), MoZh (6), NiMu (9), LKZ (5) and DaTo (10), others include YiLi (1), YaQi (2) total of 55 individuals were sequenced at a medium depth of ~10X to satisfy the genome coverage of the sequencing data. The rest of 73 individuals (9 of DeBa, 7 of NiQi, 4 of JiZi, 5 of LKZ, 7 of JiCh, 4 of PrZ, 6 of ThB, 6 of WMG, 6 of ELC, 7 of MoGo, 4 of YaQi, 8 of YiLi) were sequenced with a low depth of ~5X. With the help of this strategy, we can obtain more variants, including SNP, Indel and CNV through population-based calling.

After extraction of genomic DNA with the protocol of Promega extraction (Promega A1125, America) from the peripheral blood. The quality and integrity of DNA was examined by the A260/A280 ratio and gel electrophoresis. At least 3μg of genomic DNA was used to construct a Nano TruSeq Illumina DNA library with an insert size of 350bp following the manufacturer’s instructions (Illumina Inc.). The libraries were sequenced on Illumina HiSeq XTen/2500 instruments at BerryGenomics Company (Beijing, China) using 150/125 cycles and the paired-end mode. Detailed information about the sequencing library type and read quality is given in **Supplementary Table S2.** High-quality DNA for genome sequencing was processed to construct short-insert (350 bp) DNA libraries according to the manufacturer’s specifications (Illumina, San Diego, CA). To generate 500-bp mate-paired libraries, we used the Covaris Ultrasonic Processor (Covaris, Woburn, MA) to cut genomic DNA into 500-bp fragments randomly, followed by the process of end repairing, adding A to the tails, purification and PCR amplification. The Qubit v.2.0 kit (Life Technologies, Gaithersburg, MD) was used to analyze the quality of the constructed libraries. After diluting each library to 1 ng/μl, an Agilent 2100 Bioanalyzer (Agilent Technologies, Palo Alto, CA) was used to check the insert size of the libraries, and real time q-PCR was then performed to detect the effective concentration of the libraries. The qualified libraries with appropriate insert size (350 bp) and concentration (> 2 nM) were sequenced using the Illumina HiSeq XTen and HiSeq 2500 platform (Illumina, San Diego, CA), and 125-bp paired-end reads were generated and managed using Illumina HiSeq Control Software (HCS) v3.3. Finally, we produced approximately 3,150 Gb clean data (total of 25.87 billion raw reads).

**Mapping**

First, the reference genome (EquCab 2.0) should be indexed with the command “index” of BWA software. The paired-end clean reads were aligned to the horse reference genome using the bwa “aln” module in Burrows–Wheeler Algorithm (BWA, version: 0.7.12) with default settings. By using the “sampe” parameter of BWA, the aligned files were converted to “sam” file. Then the mapping results were converted to BAM format by SAMtools (Version: 1.1) ([Li, et al. 2009](#_ENREF_10)) and sorted by SortSam tools in Picard packages (picard.sourceforge.net, Version: 1.86) by settings of SORT_ORDER=coordinate and VALIDATION_STRINGENCY=SILENT. The SAMtools view module was applied to remove the unmapped and multi-hit reads by using settings of –h –F 4 –b and –bq 1 respectively. Mark Duplicates tool in Picard packages were used to remove duplicate reads. Only properly paired reads both aligned to the reference were retained for subsequent analysis (**Supplementary Table S2**).The BamCoverage (<https://github.com/BGI-shenzhen/BamCoverage/blob/master/README.md>) was used to compute the coverage and depth of sequence alignments, with the “statistics Coverage” parameter. The average mapping rate was 96.08% and the average depth-of-coverage was approximately 10-fold in most of the animals studied.

**SNP calling**

The Picard Mark Duplicates tool was used to remove PCR duplicates. Genome Analysis Toolkit (GATK, version 2.4) ([McCormick, et al. 2015](#_ENREF_13)) and SAMtools were used to detect SNPs following the methodology from ([Zhou, et al. 2015](#_ENREF_26)). Briefly, reads were realigned around indels using the Realigner Target Creator and Indel Realigner tools from GATK, before calling SNPs with the GATK Unified Genotyper and SAMtools mpileup modules, separately. SNPs were retained if matching the following five criteria: (1) the SNP confidence score (QD) was greater than or equal to 20; (2) the Phred-scaled P-value of the Fisher’s exact test to detect strand bias (FS) was inferior to equal to 10; (3) the Z-score of the Wilcoxon rank sum test of Alt vs. Ref read position bias (ReadPosRankSum) was greater or equal to -8; (4) the Qual score of each individual SNP was larger-than-average; (5) SNPs showed only two possible alleles and a minimal allele frequency of 5%.The 138 individual SNP VCF files were combined into the merged dataset of 10,376,152 autosomal SNPs and this merged SNP dataset was further phased to impute its own missing positions using BEAGLE software ([Browning and Browning 2007](#_ENREF_3)).

Indels were called following the methodology that was used for calling SNPs with the Unified Genotyper, excepting that the –glm option was set to INDEL to report indel only. Copy number variants were called by CNVcaller software([Wang, et al. 2017](#_ENREF_21)) (https://github.com/JiangYuLab/CNVcaller). This software is based on read-depth of clean reads and corrected by GC content **(Supplementary Figure S1)**.

**Annotation**

SNP variants were classified into protein coding regions (overlapping a coding exon), 5’UTRs and 3’UTRs (overlapping untranslated region), intronic regions (overlapping with an intron), or intergenic regions using the horse genome GTF file downloaded from Ensembl 94 (http://asia.ensembl.org/index.html) and the SNPEff software (Version: 4.0) ([Cingolani, et al. 2012](#_ENREF_4)). SNPs located within protein coding regions were further binned into synonymous and non-synonymous SNPs **(Supplementary Table S3**). We cross-validated the list of variants by identifying how many overlapped with the dbSNP database (ncbi.nlm.nih.gov/projects/SNP/) (**Supplementary Table S4)**.

**Diversity analysis**

The within-population genetic diversity for Chinese horse populations was assessed using the filtered SNPs and various metrics, including observed (H_o_) and expected heterozygosity (H_e_) **(Supplementary Table S5)**.

Linkage disequilibrium (LD) was calculated between pairs of autosomal SNPs through the LDdecay software (github.com/BGI-shenzhen/PopLDdecay). Run of homozygosity (ROHs) for each population of horse breeds, including the number of ROHs, the size per ROHs, and the total size within ROHs foreach individual, were estimated using the parameters of “--homozyg-density 1,000, --homozyg-window-het 1 --homozyg-kb 500, and --homozyg-window-snp 50” **(Supplementary Figure S2)**.

**Phylogenetic analysis**

All SNPs were pruned using PLINK (Version:1.90b) and considering window sizes of 1,000 variants, a step size of 5, and a pairwise r^2^ threshold of 0.5 (--indep-pairwise 1000 5 0.5). This retained a total of 1,449,645 independent SNPs for all subsequent analyses. The principal component analysis (PCA) was carried out using the GCTA 1.91 software ([Xu, et al. 2011](#_ENREF_23)). For the first PCA plot all 138 animals were used, with the first three principal components cumulatively explaining 11.95% of the total variance. Population structure was evaluated using ADMIXTURE (Version:1.3.0)([Alexander, et al. 2009](#_ENREF_1)), considering a number of 10,000 iterations and two to six genetic clusters (*K*). The neighbor-joining tree ([Saitou and Nei 1987](#_ENREF_19)) was constructed using PHYLIP 3.68 ([evolution.genetics.washington.edu/phylip.html](http://evolution.genetics.washington.edu/phylip.html)). MEGA5 ([Tamura, et al. 2011](#_ENREF_20)) and FigTree software (tree.bio.ed.ac.uk/software/figtree/) were used to visualize the phylogenetic trees. The sequence alignment underlying the phylogenetic tree presented on **Figure 1D** is available for downloading from the ftp website.

Besides, we have tried to draw the phylogeographic trees by using four-fold degenerate nucleotide sites to explore demographical history by the software Beast v1.10 ([Drummond and Rambaut 2007](#_ENREF_6)), a program used for Bayesian Markov chain Monte Carlo analysis of genetic sequences. BEAST was run on the dataset using a log-normal relaxed clock model, for 100,000,000 states to generate the maximum clade credibility (MCC) tree. Each analysis was run in triplicate with the first 10,000,000 states discarded as the burn-in period. We used the HEK_GAMMA model of nucleotide substitution, with four rate categories for gamma-distributed rates across sites. Convergence was assessed in Tracer (REF) using effective sample size (ESS) of at least 598. The MCC tree returned from BEAST was then visualized using SpreaD3 ([Bielejec, et al. 2011](#_ENREF_2)), where the size of the circles around each sampling location is proportional to the number of lineages maintained at that location and the colors stand for the different horse populations (**Supplementary Figure S6**).

***∂a∂i***

We refined the multiple demographic scenarios related to horses domestication using a diffusion approximation method for the allele frequency spectrum ∂a∂i([Gutenkunst, et al. 2009](#_ENREF_9)). This program estimates demographic parameters based on the diffusion approximation to the site frequency spectrum (SFS). This can also show statistically significant support for population models including migration over models without migration. First, to minimize potential effects of selection that could interfere with demographic inference, we limited our analyses to the SNPs present within the noncoding regions across the whole genome regions. Second, the folded allele frequency spectrum for the three populations (NC, SW and QT horse breeds) was projected down in ∂a∂i. We tested four different scenarios to reconstruct the demographic history of the domesticated breeds of horses (**Supplementary Table S6**), and the model with the highest log-likelihood value was considered to be optimal.

Model 1: The lineage ancestral to the Northern horse breeds first split from a lineage ancestral to the remaining breeds; the latter further split into a lineage ancestral to the Southern horse breeds and the Qinghai-Tibetan horse breeds;

Model 2: The lineage ancestral to the Southern horse breeds first split from a lineage ancestral to the remaining breeds; the latter further split into a lineage ancestral to the Northern horse breeds and the Qinghai-Tibetan horse breeds;

Model 3: The lineage ancestral to the Qinghai-Tibetan horse breeds first split from a lineage ancestral to the remaining breeds; the latter further split into a lineage ancestral to the Northern horse breeds and the Southern horse breeds;

Model 4: A single radiation gave simultaneously rise to the ancestors of the Northern breeds, the Southern breeds, and the Qinghai-Tibetan horse breeds, respectively;

As suggested, we specified simple models first (without migration) and gradually fit the model with increasing complexity. To get the best simple model, we used ∂a∂i and also confirmed by fastsimicoal2 software ([Excoffier and Foll 2011](#_ENREF_7)). First, we use ∂a∂i to run the program 20 times with varying starting points to ensure convergence and retained the fitting with the highest likelihood. Then, we used fastsimicoal2 with the following parameters:“-M 0.0001 -n100000 -N100000 -l 10 -L 40 -q --multiSFS -C 5 -c 8 -m” as suggested by ([Liu, et al. 2018](#_ENREF_11)). For each model, 50 repeat runs were carried out to test the best model and the additional code is shown below. Then we found Model4 with the highest likelihood (**Supplementary Table S7**). Then, we used a strategy in which a newly added parameters related with migration and population size was discarded if it did not bring a marked improvement to the model log likelihood and the Akaike information criterion (AIC) ([Yang, et al. 2016](#_ENREF_24)). Gene flow was modeled as discrete migration events at a certain time after population divergence. After model selection, scaled parameters for the best-supported model were transformed into the real values using the values reported by ([Orlando, et al. 2013](#_ENREF_15)) for the mutation rate (μ=7.242e-9) and the generation time (g=8 years).

The Python function used to estimate the final model parameters in ∂a∂i is as

follows:

def split_asymmig_all(params, ns, pts):

"""

Model with split between pop 1 and (2,3), then split between 2 and 3.

Migration is symmetrical between all population pairs (ie 1<->2, 2<->3, and 1<->3).

"""

#11 parameters

nuA, nu1, nu2, nu3, m12, m21, m13, m31, m23, m32, T = params

xx = Numerics.default_grid(pts)

phi = PhiManip.phi_1D(xx)

phi = Integration.one_pops(phi, xx, T1=0, nu=nuA)

phi = PhiManip.phi_1D_to_2D(xx, phi)

phi = PhiManip.phi_2D_to_3D_split_2(xx, phi)

phi = Integration.three_pops(phi, xx, T, nu1=nu1, nu2=nu2, nu3=nu3, m12=m12, m21=m21, m23=m23, m32=m32, m13=m13, m31=m31)

fs = Spectrum.from_phi(phi, ns, (xx,xx,xx))

return fs

fastsimicoal2 code

Model1.tpl

Model1.est

//Parameters for the coalescence simulation program : fsimcoal2.exe

3 Samples to simulate :

//Population effective sizes (number of genes)

NPOPNC

NPOPSW

NPOPQT

//Samples sizes and samples age

20

20

20

//Growth rates : negative growth implies population expansion

0

0

0

//Number of migration matrices : 0 implies no migration between demes

0

//historical event: time, source, sink, migrants, new deme size, new growth rate, migration matrix index

2 historical event

TDIV1 1 0 1 RESIZE1 0 0

TDIV2 2 1 1 RESIZE2 0 0

//Number of independent loci [chromosome]

1 0

//Per chromosome: Number of contiguous linkage Block: a block is a set of contiguous loci

1

//per Block:data type, number of loci, per generation recombination and mutation rates and optional parameters

FREQ 1 0 7.242e-9 OUTEXP

// Priors and rules file

// *********************

[PARAMETERS]

//#isInt? #name #dist.#min #max

//all Ns are in number of haploid individuals

1 ANCSIZE unif 1000 100000 output

1 NPOPdom unif 10 10000 output

1 NPOPNC unif 10 10000 output

1 NPOPSW unif 10 10000 output

1 NPOPQTunif 10 10000 output

1 TDIV1 unif 10 10000 output

1 TDIVPLUS unif 10 10000 output

[RULES]

[COMPLEX PARAMETERS]

0 RESIZE1 = ANCSIZE/NPOPNorth hide

0 RESIZE2 = NPOPdom/NPOPPony hide

Model2.tpl

// Priors and rules file

// *********************

[PARAMETERS]

//#isInt? #name #dist.#min #max

//all Ns are in number of haploid individuals

1 ANCSIZE unif 1000 100000 output

1 NPOPWP unif 10 10000 output

1 NPOPNC unif 10 10000 output

1 NPOPSW unif 10 10000 output

1 NPOPQTunif 10 10000 output

1 TDIV1 unif 10 10000 output

1 TDIVPLUS unif 10 10000 output

[RULES]

[COMPLEX PARAMETERS]

0 RESIZE1 = ANCSIZE/NPOPSW hide

0 RESIZE2 = NPOPWP/NPOPNC hide

Model2.est

//Parameters for the coalescence simulation program : fsimcoal2.exe

3 Samples to simulate :

//Population effective sizes (number of genes)

NPOPNC

NPOPSW

NPOPQT

//Samples sizes and samples age

20

20

20

//Growth rates : negative growth implies population expansion

0

0

0

//Number of migration matrices : 0 implies no migration between demes

0

//historical event: time, source, sink, migrants, new deme size, new growth rate, migration matrix index

2 historical event

TDIV1 0 1 1 RESIZE1 0 0

TDIV2 2 0 1 RESIZE2 0 0

//Number of independent loci [chromosome]

1 0

//Per chromosome: Number of contiguous linkage Block: a block is a set of contiguous loci

1

//per Block:data type, number of loci, per generation recombination and mutation rates and optional parameters

FREQ 1 0 7.242e-9 OUTEXP

Model3.est

// Priors and rules file

// *********************

[PARAMETERS]

//#isInt? #name #dist.#min #max

//all Ns are in number of haploid individuals

1 ANCSIZE unif 1000 100000 output

1 NPOPWL unif 10 10000 output

1 NPOPNCunif 10 10000 output

1 NPOPSW unif 10 10000 output

1 NPOPQTunif 10 10000 output

1 TDIV1 unif 10 10000 output

1 TDIVPLUS unif 10 10000 output

[RULES]

[COMPLEX PARAMETERS]

0 RESIZE1 = ANCSIZE/NPOPQT hide

0 RESIZE2 = NPOPWP/NPOPNC hide

Model3.tpl

//Parameters for the coalescence simulation program : fsimcoal2.exe

3 Samples to simulate :

//Population effective sizes (number of genes)

NPOPNC

NPOPSW

NPOPQT

//Samples sizes and samples age

20

20

20

//Growth rates : negative growth implies population expansion

0

0

0

//Number of migration matrices : 0 implies no migration between demes

0

//historical event: time, source, sink, migrants, new deme size, new growth rate, migration matrix index

2 historical event

TDIV1 0 2 1 RESIZE1 0 0

TDIV2 1 0 1 RESIZE2 0 0

//Number of independent loci [chromosome]

1 0

//Per chromosome: Number of contiguous linkage Block: a block is a set of contiguous loci

1

//per Block:data type, number of loci, per generation recombination and mutation rates and optional parameters

FREQ 1 0 7.242e-9 OUTEXP

Model4.est

//Parameters for the coalescence simulation program : fsimcoal2.exe

3 Samples to simulate :

//Population effective sizes (number of genes)

NPOPNC

NPOPSW

NPOPQT

//Samples sizes and samples age

20

20

20

//Growth rates : negative growth implies population expansion

0

0

0

//Number of migration matrices : 0 implies no migration between demes

0

//historical event: time, source, sink, migrants, new deme size, new growth rate, migration matrix index

3 historical event

TDIV 1 0 1 1 0 0

TDIV 2 0 1 1 0 0

TDIV 0 0 0 RESIZE1 0 0

//Number of independent loci [chromosome]

1 0

//Per chromosome: Number of contiguous linkage Block: a block is a set of contiguous loci

1

//per Block:data type, number of loci, per generation recombination and mutation rates and optional parameters

FREQ 1 0 7.242e-9 OUTEXP

Model4.tpl

// Priors and rules file

// *********************

[PARAMETERS]

//#isInt? #name #dist.#min #max

//all Ns are in number of haploid individuals

1 ANCSIZE unif 1000 100000 output

1 NPOPQT unif 10 10000 output

1 NPOPSW unif 10 10000 output

1 NPOPNC unif 10 10000 output

1 TDIV unif 10 10000 output

[RULES]

[COMPLEX PARAMETERS]

0 RESIZE1 = ANCSIZE/NPOPNC hide

Note: “NPOPNC”, “NPOPSW”, “NPOPQT” means the population effective size, which will be defined in the “est” file. Historical event: means time, source, sink, migrants, new deme size, new growth rate, migration matrix index; Per chromosome: Number of contiguous linkage Block: a block is a set of contiguous loci with the default data 1.

Subsequently, we calculated f3-statistics ([Patterson, et al. 2012](#_ENREF_16)) using the ‘threepop’ program (-k 5000) provided by the TreeMix package. Significantly negative f3 (C; A, B) statistics suggest that population C can be modelled as a mixture of populations A and B **(Supplementary Table S8)**. D-statistics ([Green, et al. 2010](#_ENREF_8)) were estimated to assess patterns of shared derived alleles across breeds using the qpDstat software in Admixtools ([Green, et al. 2010](#_ENREF_8)) **(Supplementary Table S9)**.

**Selective sweeps**

We divided all the populations to Qinghai-Tibetan Plateau group (QT) and Lowland group (LL). The QT group include LKZ, JiZi, NiMu, MoZh, CDM, DaTo and QiLi populations, total of 55 individuals, and the LL group include DeBa, NiQi, JiCh, MoGo, WMG, ELC, YiLi and YaQi, total of 65 individuals. We performed a genomic scan by combing three selection signature tests of the population-differentiation statistic (F_ST_) ([Weir and Cockerham 1984](#_ENREF_22)), the transformed heterozygosity score (ZH_P_) and the relative nucleotide diversity (θ_π_ ratio, θ_π -LL_/θ_π -QT_ ) ([Nei and Li 1979](#_ENREF_14)) of the LL to the QT. F_ST_ and nucleotide diversity (θ_π_) were calculated by VCFTools ([Danecek, et al. 2011](#_ENREF_5)). The window-based ZHp approach was calculated as previously described ([Rubin, et al. 2012](#_ENREF_18)). In brief, *H*_p_ = 2Σ*n*_MAJ_Σ*n*_MIN_/(Σ*n*_MAJ_ + Σ*n*_MIN_)^2^, where Σ*n*_MAJ_ is the sum of major allele frequencies within a sliding window, and Σ*n*_MIN_ is the sum of minor allele frequencies in the same window. The individual *H*_p_ values were Z transformed as follows: ZH_p_ = (*H*_p_ - *μH*_p_)/*σH*_p_, where *μ* is the overall average heterozygosity and *σ* is the standard deviation of all windows within each group([Rubin, et al. 2012](#_ENREF_18)). The θ_π_ ratio was computed of the low-altitude horse breeds to the high-altitude. We consider top 5% level for empirical percentile (F_ST_>0.02, θπ ratio>1.09, |ZH_P_| >1.88) windows as candidate outliers in strong selective sweeps. To annotation candidate genes harbored in these selective regions, we used Rscript to map genes in selective windows.

The overlapping windows shared by all three tests were considered as conservative candidate selection targets and were further annotated by the genomic database BioMart (http://www.biomart.org/). The Gene Ontology (GO) enrichment analysis of the annotated candidates were performed by using both the online G: profiler **(Supplementary Table S14)**.

**Validation in the extended population**

Kompetitive Allele Specific PCR (KASP) genotyping platforms was used to test the three mutations, *EPAS1* gene (R144C, SNP1; E263D, SNP2) and the *HBE1* gene (V147A, SNP3). Primers were EPAS1-R144C-FAM: CATTTTTGAGACTCAGGTTCTCACG; EPAS1-R144C-HEX: CCATTTTTGAGACTCAGGTTCTCACA; EPAS1-R144C-common: ATCCCTGTGACCATGAGGAGATC (FAM for G, HEX for A) and HBE1-V76A-FAM: CTGACCTCTTTTGGAGATGCTGC; HBE1-V76A-HEX: GCTGACCTCTTTTGGAGATGCTGT. The EPAS1-E263D mutation (SNP3) was genotyped in 908 horses of the extended dataset by using ligation detection reaction (LDR) ([Yi, et al. 2009](#_ENREF_25)) with primers: EPAS1-E263D-F:AACTTACGGTTCTGGTGACTTT, EPAS1-E263D-R: TCTGGAGTGTGTTCATCTGAGT, product length: 230 bp.

**Cell maintenance**

A549 cells and HepG2 cells were incubated at 37℃ with 5% CO_2_ in Cell thermostat carbon dioxide culture box. A549 cells represent adenocarcinoma human alveolar basal epithelial cells and were selected in this study for their higher expression levels compared to other available cell types ([Prabhakar and Semenza 2012](#_ENREF_17)). HepG2 cell is Human hepatocellular liver carcinoma cell line.

**Transfection**

Transfection of the recombinant plasmids into A549 cells was carried out by using Lipofectamine3000 (Invitrogen, America). First, 3.75 μL of Lipofectamine3000 Reagent (Invitrogen, America) and 2.5 μL of Endofree plasmid were diluted in 125 μL of Opti-MEM® I Reduced Serum medium, GlutaMAX™ (Thermo Fisher Scientific, USA). This solution was then mixed with Lipofectamine plasmids Reagent (1:1 ratio) and incubated for 15 min at room temperature, before it was added to 6-well plates (Costar, USA) showing 30–50% confluent cells. Then cells were incubated at 37℃ with 5% CO_2_ about 48 hours. The same dose of lipofectamine was used for co-transfection of EPAS1- and ARNT- plasmids into A549 cells to generate cells co-expressing myc-tagged EPAS1 and flag-tagged ARNT proteins for Co-IP experiment.

**Western blotting**

Cells were harvest-washed with PBS, before they were homogenized with a Dounce glass homogenizer using ice-cold Mammalian Protein Extraction Reagent (78501, Thermo Fisher) supplemented with protease inhibitors (Roche). Following 10 minutes on ice, cell lysates were centrifuged at 14,000 g for 20 min at 4℃, and supernatants were collected. The total amount of proteins was measured with the BCA (Biocinchoninic acid) Protein Assay Kit (Thermo Fisher, America). Additionally, cell lysates were electrophoresed on SDS-PAGE under reducing conditions with Tris-glycine buffer (pre-running = 15 min, running = 55 min) and transferred to a nitrocellulose membrane (Bio-Rad) following standard procedures. Membranes were blocked with 5% nonfat dried milk and then incubated with anti-GFP (Abmart) and β-tubulin (CST, USA) for 2 hours. After washing in 1X TBST for 10 min 3 times, membranes were incubated with secondary antibodies anti-mouse antibody (Abmart) and anti-rabbit antibody (CST, USA) for 1 hour, respectively. Antigen-antibody complexes were visualized by enhanced chemiluminescence detection (ECL, Thermo Fisher) to measure the protein amount of GFP-tagged EPAS1 protein.

**qPCR quantification**

After washing in PBS, cells were harvested, and total RNA was extracted using RNA extraction Kit (Promega) according to the manufacturer’s instructions. RNA was reverse transcribed with PrimeScript TR reagent Kit (Takara). Quantitative Real-Time PCR analysis of the *VEGFA, VHL* and *LDHA* were carried out using the ABI7500 sequence detection system (Applied Biosystems by Life Technologies, Darmstadt, Germany). The ubiquitous *β-actin* gene served as a reference control *(http://icg.big.ac.cn/index.php/Homo_sapiens#Internal_Control_Genes_3)*. For gene *Erythropoietin (EPO)* is only expressed in HepG2 cell, so we conducted transfection and RT-PCR in HepG2 cells. PCR was prepared using the Power SYBR Green PCR reagent kit (Applied Biosystems). PCR primers are shown in **Supplementary Table S18** and qPCR conditions were as follows: initial denaturation at 95 °C for 3 min, 40 cycles of denaturation at 95 °C for 15 s, with combined annealing and extension at 60 °C for 60 s. Three biological replicates were performed per sample, and the average value was used for further analysis. Fold expression changes were determined using a standard 2^-ΔΔCT^ method that compares C_T_ (cycle threshold) values of a reference gene to the gene of interest for the ΔC_T_ calculation and compares the ΔC_T_ value of a reference sample with the sample of interest for the ΔΔC_T_ calculation ([Livak and Schmittgen 2001](#_ENREF_12)).

**
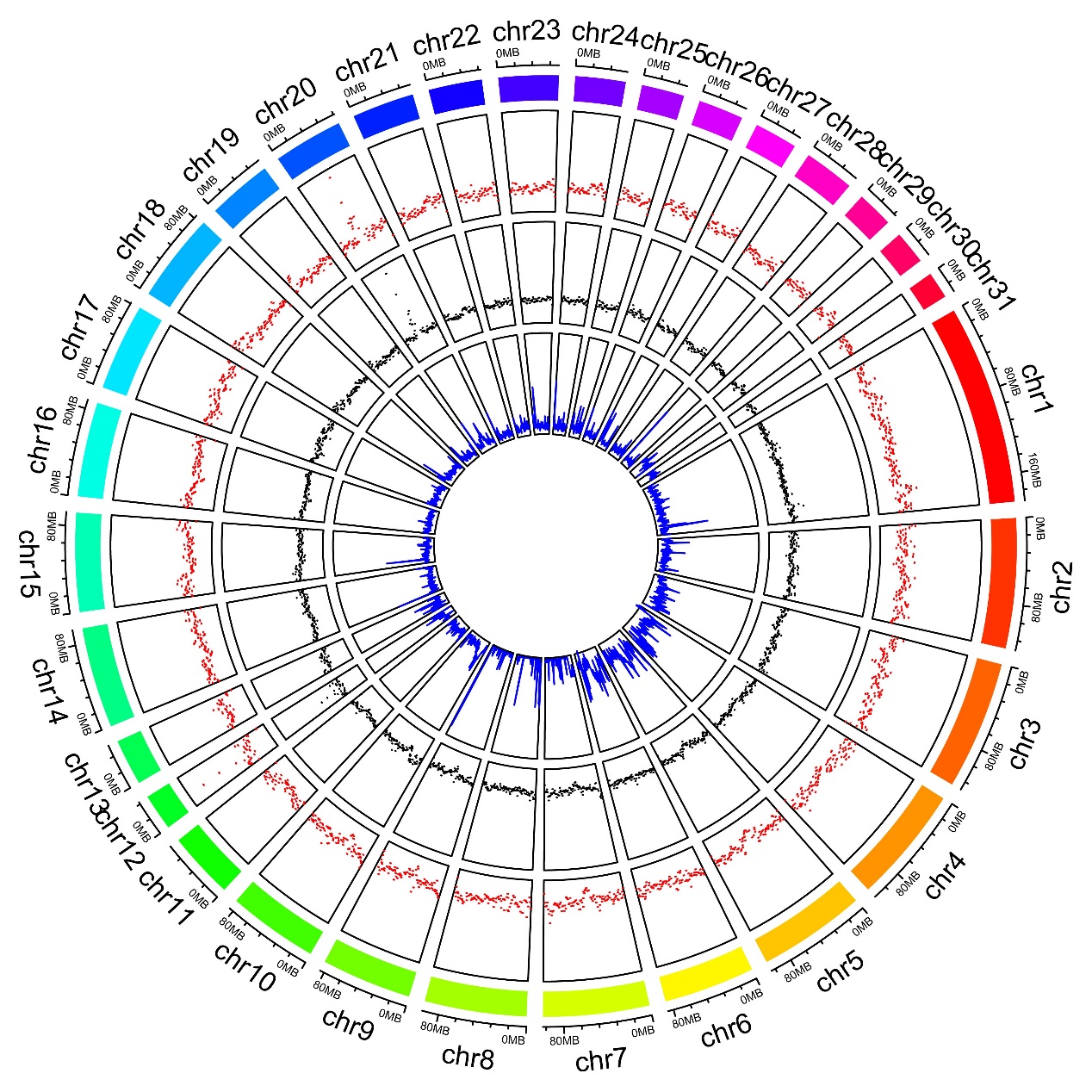
 Figure S1 Distribution of SNPs (red), INDELS (black) and CNVs (blue) on 31 chromosomes across the horse genome.**

**
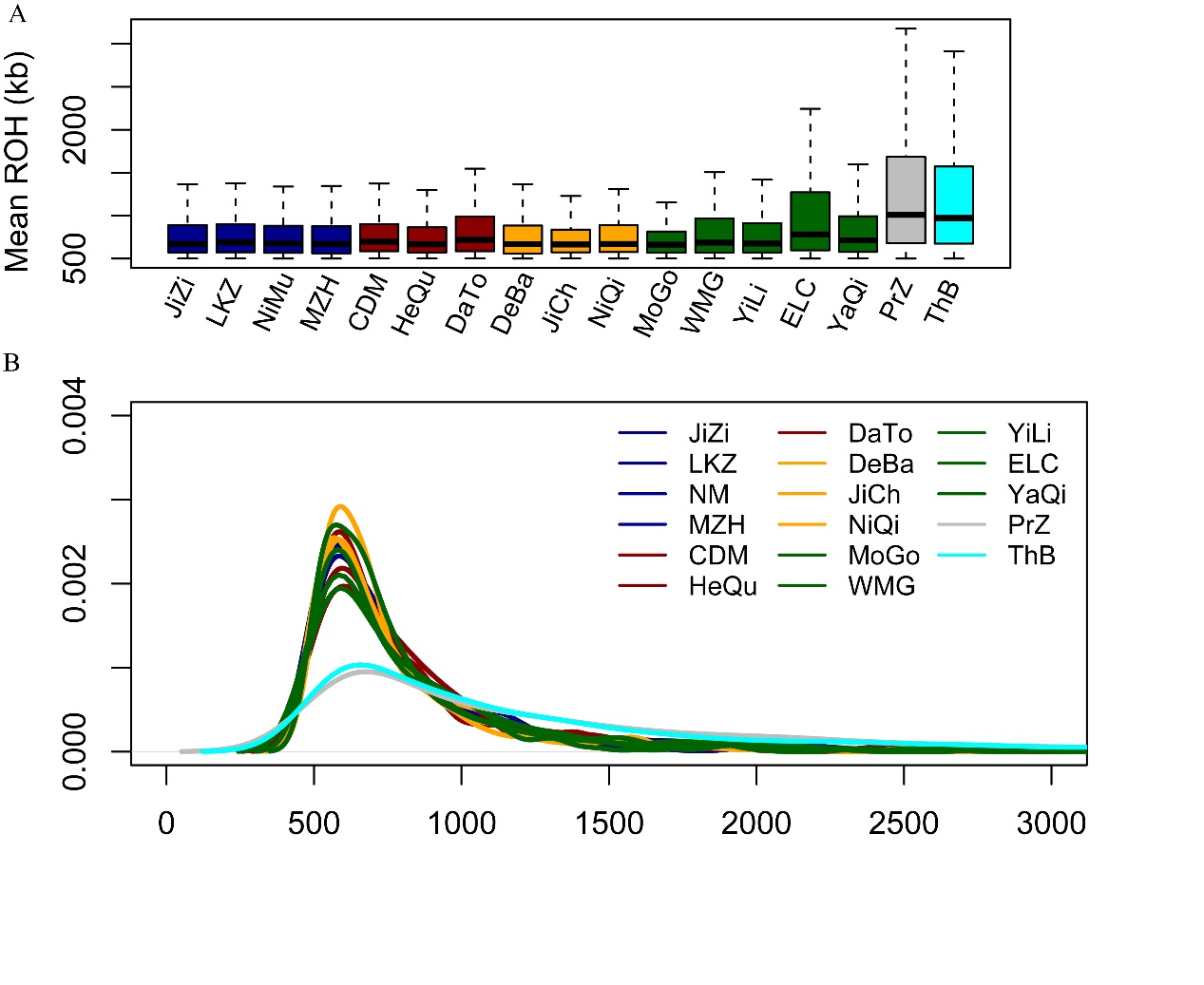
**

**Figure S2 Distribution of mean size (A) and density (B) of runs of homozygosity (ROH)** in the wild horse (gray), Thoroughbred horse (crayon) and 15 Chinese native horse breeds. The dark red color represents Tibetan horses (JiZi, Jiangzi; LKZ, Langkazi; MZH, Mozhu; NiMu, Nimu) residing at altitudes of >4,000 meters above the sea level (m.a.s.l.) and the dark blue represents Qinghai horses (CDM, Chaidamu; HeQu, Hequ; DaTo, Datong) residing at >3,000 m.a.s.l. altitude. The orange color represents the Southwestern horses (DeBa, Debao pony from Guangxi; JiCh, Jianchang pony from Sichuan; NiQi, Ningqiang pony from Xiaanxi). The dark green represents the Northern horses (MoGo, Mongolian horse from Inner Mongolia; WMG, Mongolian horse from Mongolia; ELC, ErlunChun horse from Heilongjiang; YaQi, Yanqi horse from Xinjiang; Yili horse from Xinjiang). **
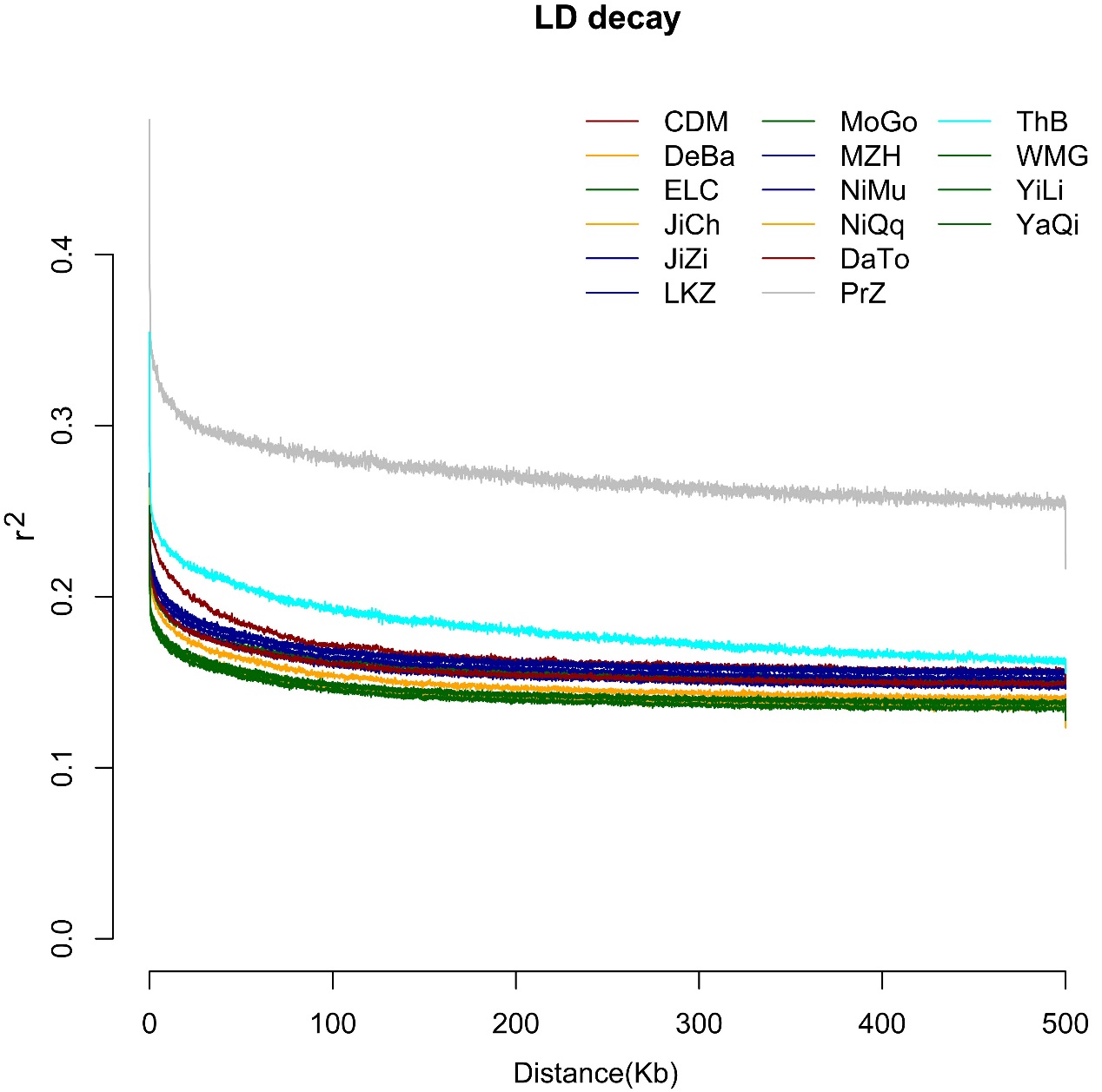
**

**Figure S3 Decay of linkage disequilibrium (LD) in the horse genome for each breed.** The colors representing populations are the same as in **Figure S2**.

**
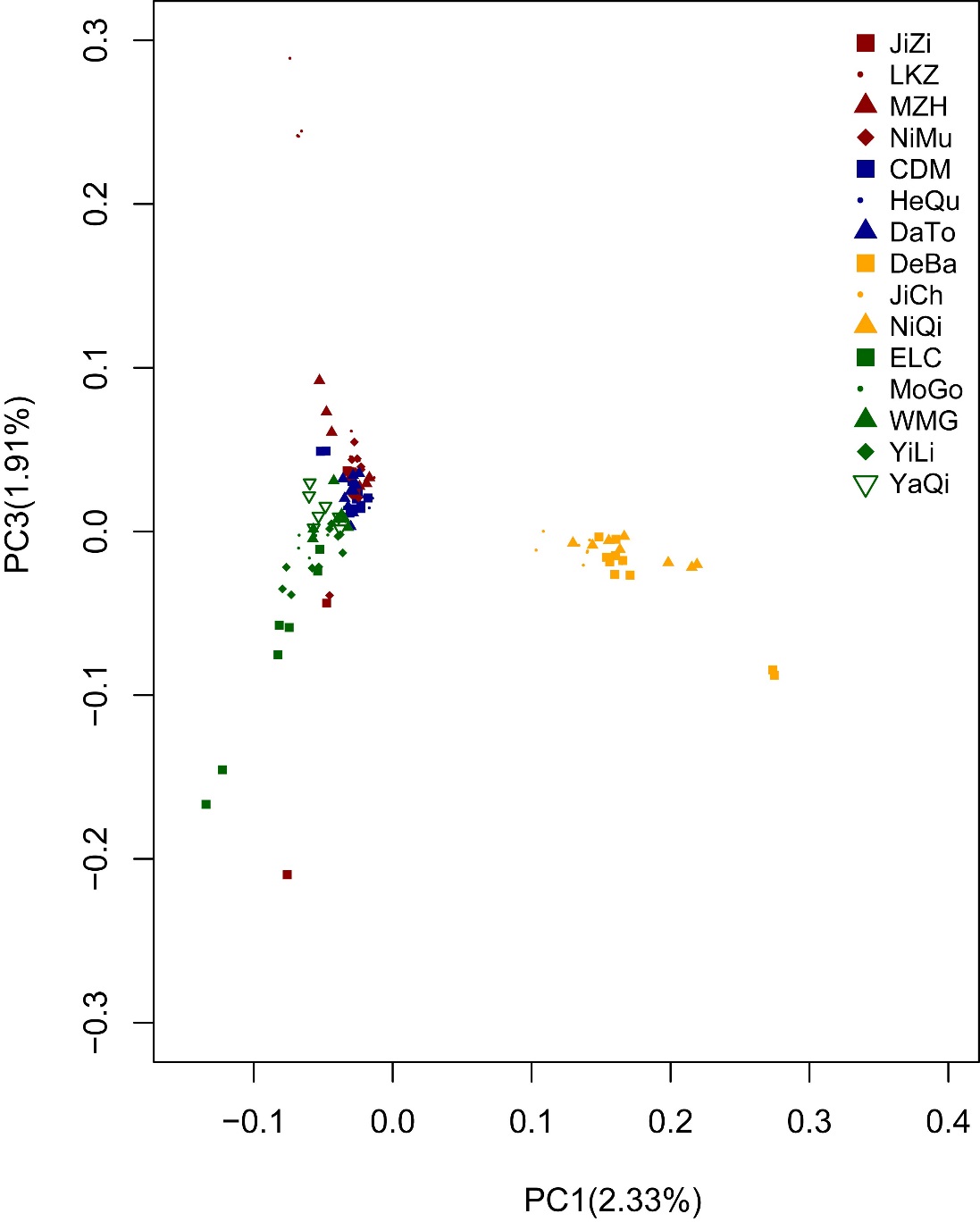
**

**Figure S4 PCA result of the first and third components of 126 Chinese native horses.** The colors representing populations are same with **Figure S2**.

**
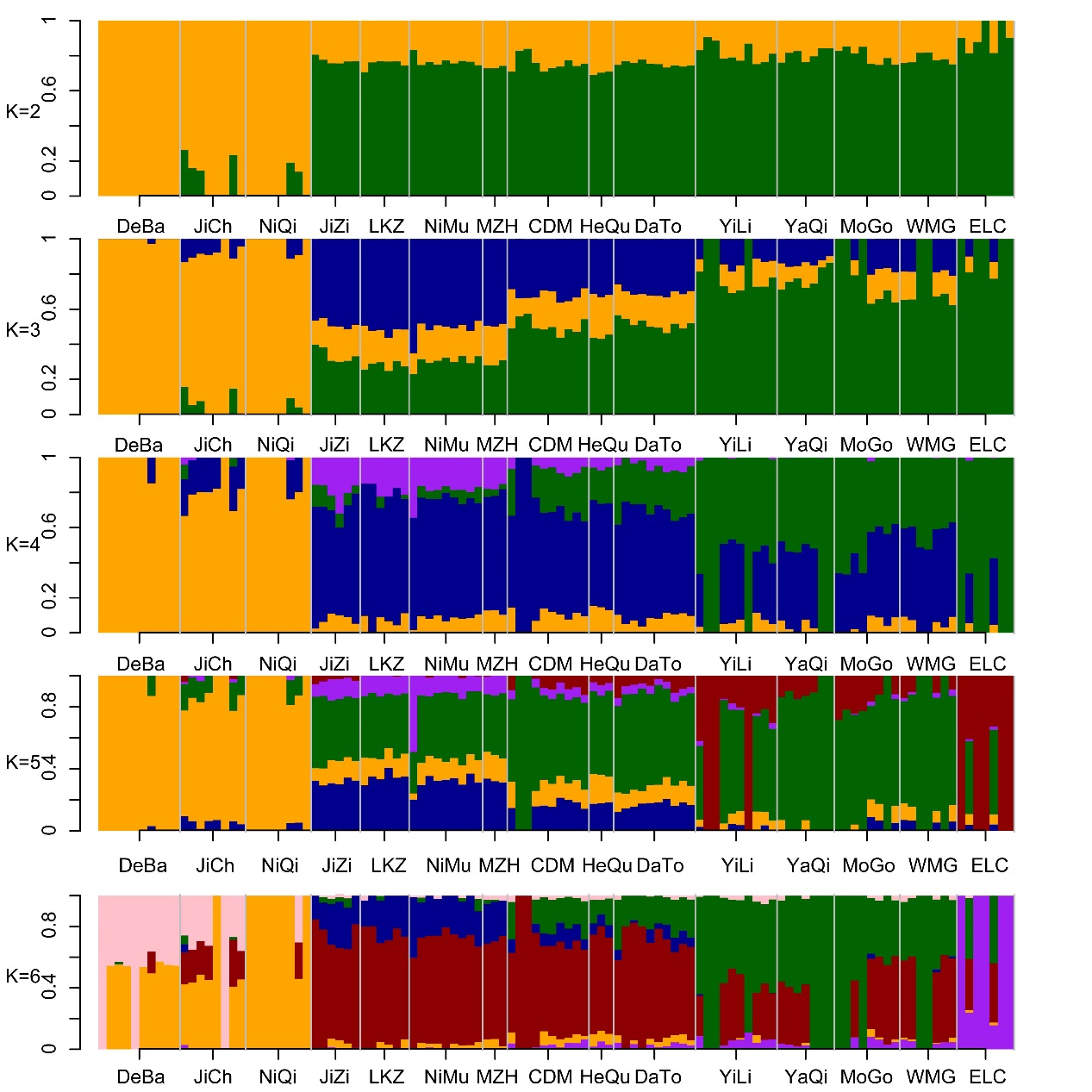
**

**Figure S5 Genetic population structure of the 126 Chinese native horses conducted by Admixture.** The length of each colored segment represents the proportion of the individual genome inferred from ancestral populations (K = 2–6).

**
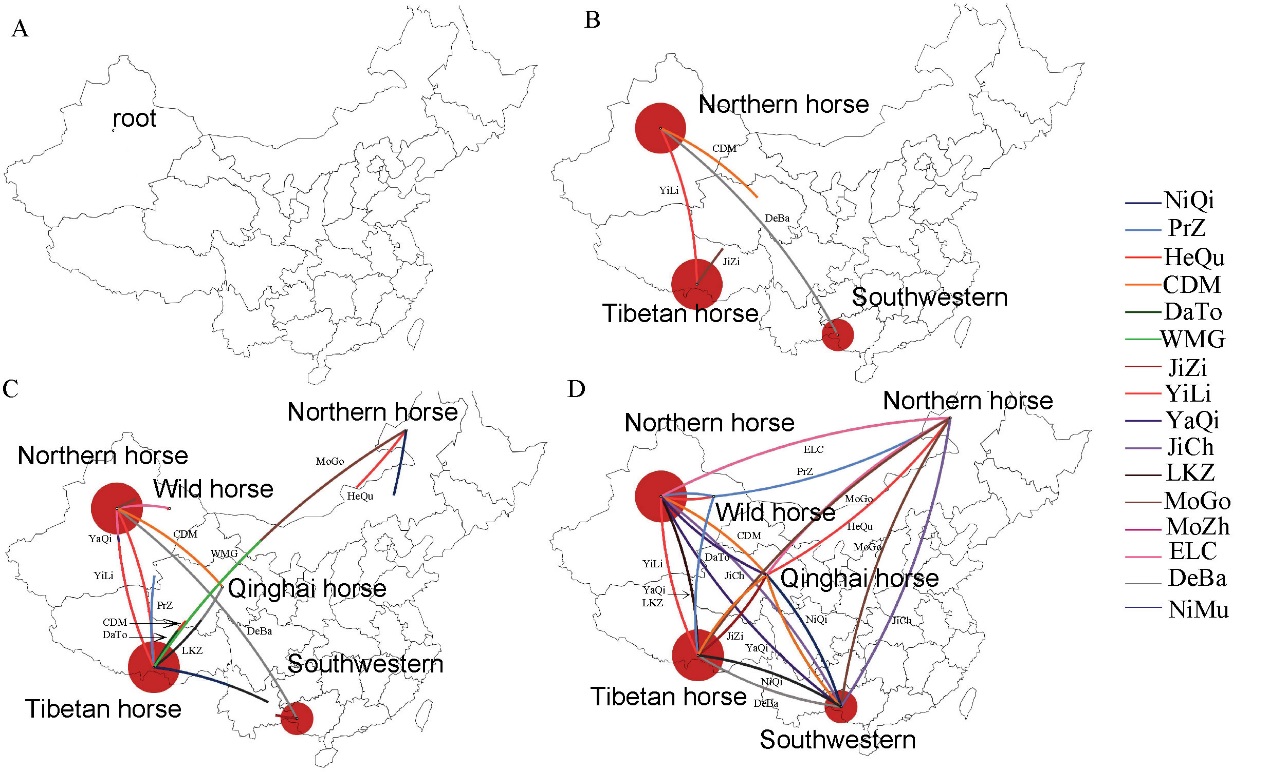
**

**Figure S6 Phylogeography distribution of Chinese horse breeds.** A-D stands for the four represented scenarios during the temporal spreading of each horse population. The MCC tree returned from BEAST was then visualized using SpreaD3. A shows the emergence of horse ancestry population denoted by root. B shows it quickly spread into three major populations, QT, SW and NC, denoted by three red circles respectively. The size of the circles around each sampling location is proportional to the number of lineages maintained at that location. C demonstrates the migration links (colored lines) occurred among three major clades (red circles). The color scheme chosen refers to the different horse populations, with reference to their parental locations. One line showing two or three colors indicates that two or more breeds from the same region belong to this lineage. To improve readability, we now show each colored line with the associated breed(s) name(s) beside. D continuously demonstrates the migration links among the Chinese horse breeds, especially between QT and SW or between NC and QT.

**_
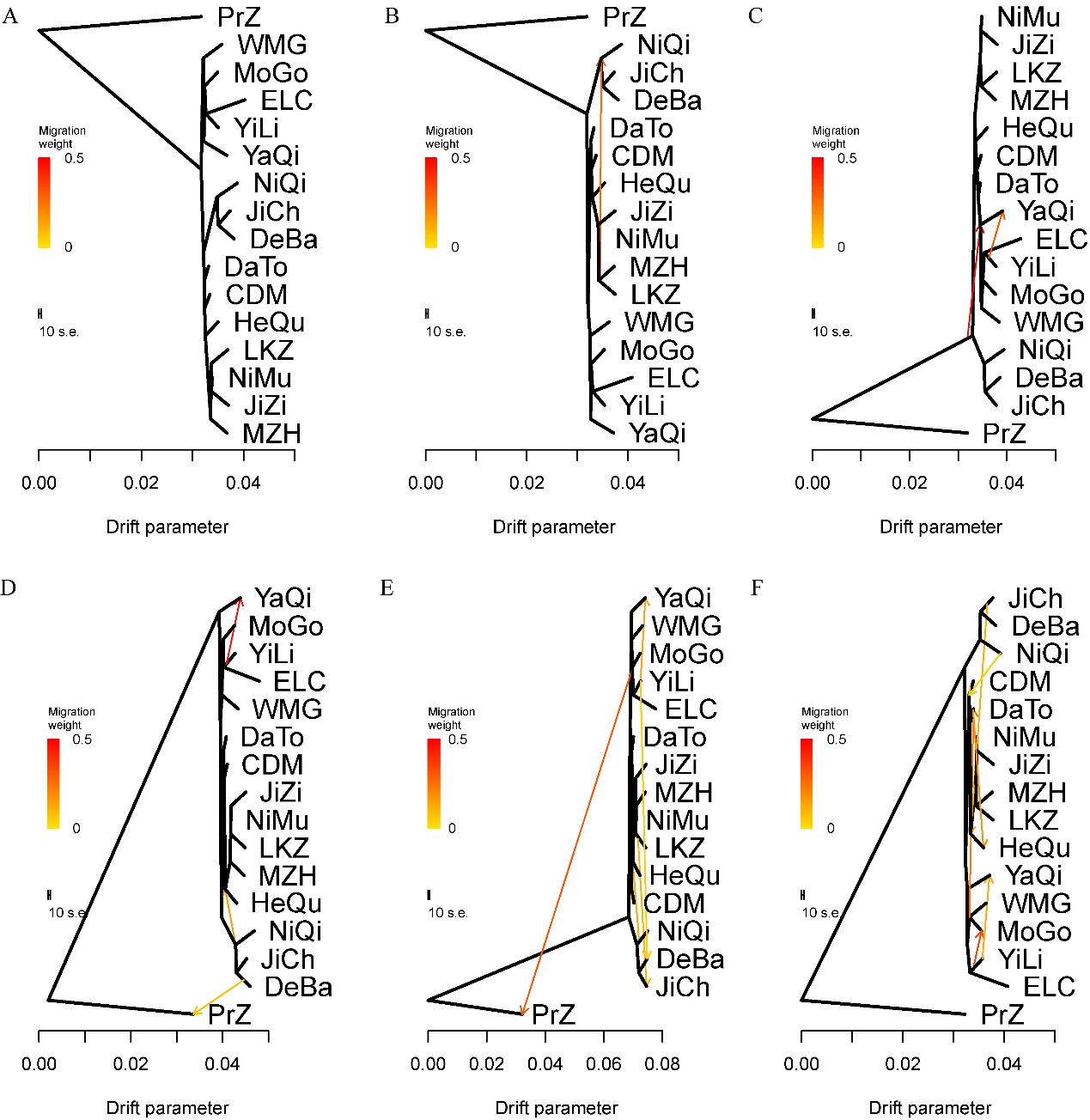
_**

**Figure S7 Migration analysis of Chinese native horse breeds by Treemix software.** (A-F) panels represent models of population affinities assuming 0-3 and 5-6 migration edges in TreeMix, respectively. The inferred migration weight is provided by the color of the arrow displayed.

**
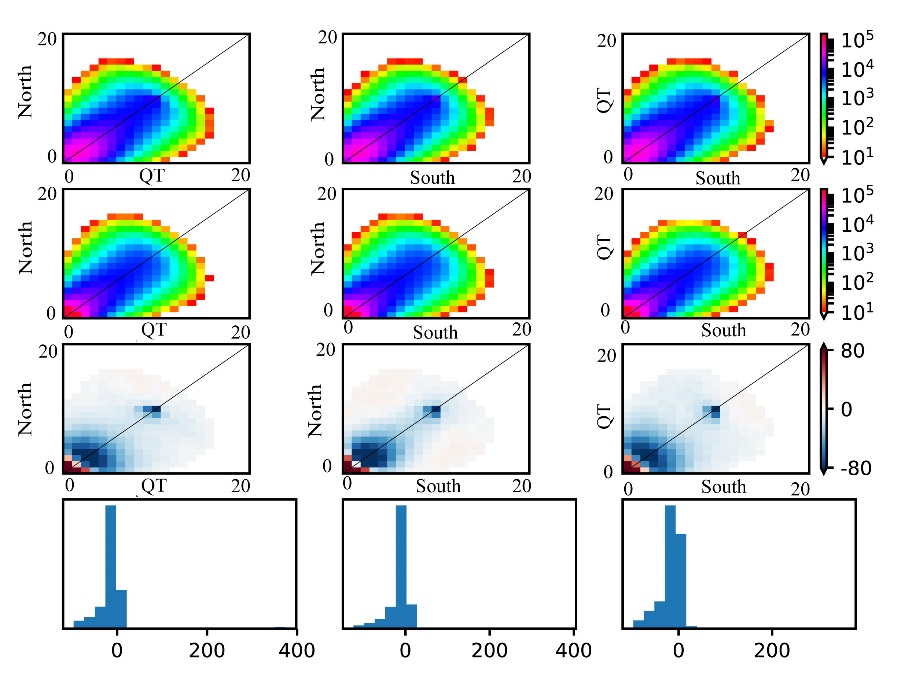
**

**Figure S8 Site frequency spectrum for the three populations of NC, SW and QT.** The frequency spectrum is shown for the data (first row) and for the best fit model (second row). The last two rows show the normalized difference (i.e. residuals) between model and data for each bin in the spectrum.

**
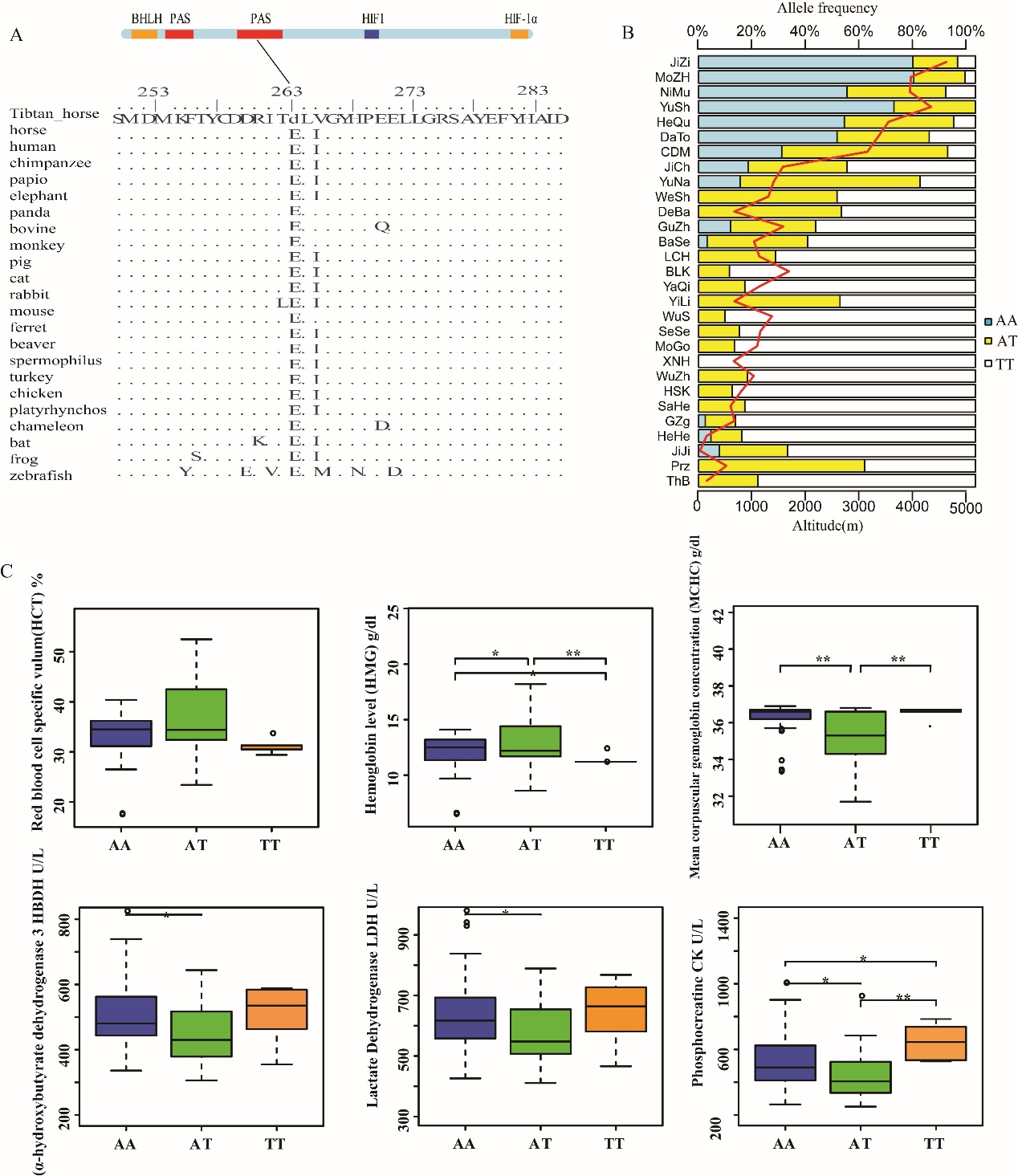
**

**Figure S9 Annotation and validation of the E263D missense SNP under positive selection in *EPAS1*.** (**A**) EPAS1 protein sequence analysis. The protein coordinates are based on the ENSECAT00000015683.1 Ensembl protein. The upper panel shows the Pfam domains of the EPAS1 protein. The two red boxes represent the Per-Arnt-Sim (PAS) domain and the yellow box represents the basic helix-loop-helix (HLH) domain. The blue box represents the hypoxia-inducible (HIF) domain and the dark orange represents the C-terminal transactivation domain (CTAD). The orthologous protein sequences from 22 vertebrates are aligned with the mutant residues shown in the box. Horse, ENSECAG00000012861; human, ENSG00000116016; chimpanzee, ENSPTRG00000023749; Papio, ENSPANG00000008730; elephant, ENSLAFG00000012; panda, ENSAMEG00000001090; bovine, ENSBTAG00000021706; monkey, ENSMFAG00000039129; pig, ENSSSCG00000008443; cat, ENSFCAT00000013633; rabbit, ENSOCUG00000014595; mouse, ENSMUSG00000024140; ferret, ENSMPUG00000002720; beaver, A0A250YLN2 (uniport); Spermophilus, ENSSTOG00000009817; turkey, ENSMGAG00000010293; chicken, ENSGALG00000036043; platyrhynchos, ENSAPLG00000010653 ; chameleon, H9G8J6 (uniprot); bat, ENSMLUG00000007567; frog, Q6GQ12 (uniprot); zebrafish，ENSDARG00000057671. (**B**) The spectrum of allele frequencies. Genotypes were determined by KASP technology at the missense polymorphism of *EPAS1* in the wild horses, Thoroughbred horses and 29 Chinese native horse breeds (N = 908 horses). The region and altitude information are shown red line. (**C**) Association analysis of the V430 SNP (mutant allele, A; reference allele, G) with the individual hemoglobin level and blood physiological values in Hequ and GZg horses. The ANOVA F-test was performed, and we found a significant association between the genotypes and HCT, HMG, MCHC, LDH, HBDH and CK. ** and * displayed the statistical significance of *P-value* <0.01 and 0.05 according to the ANOVA F-test.

**
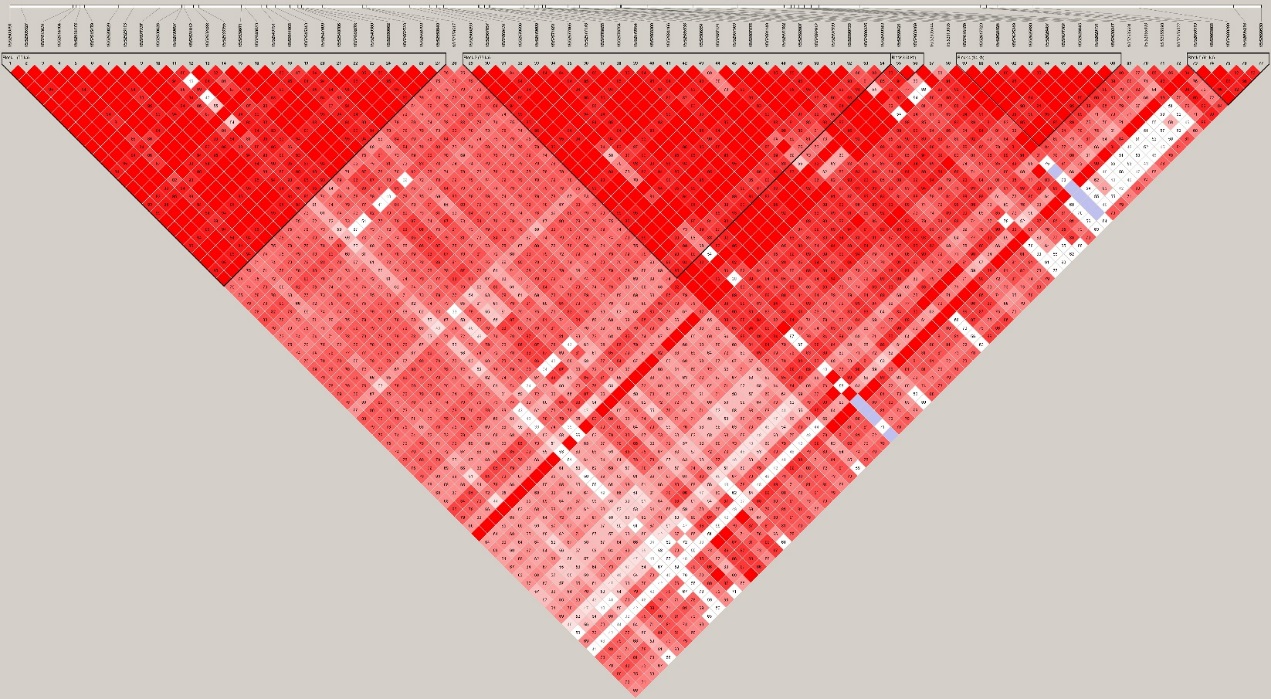
**

**Figure S10 Linkage plot of Tibetan horses in gene *EPAS1*.** The first black arrow is the E263D loci, and the second is R144C loci.


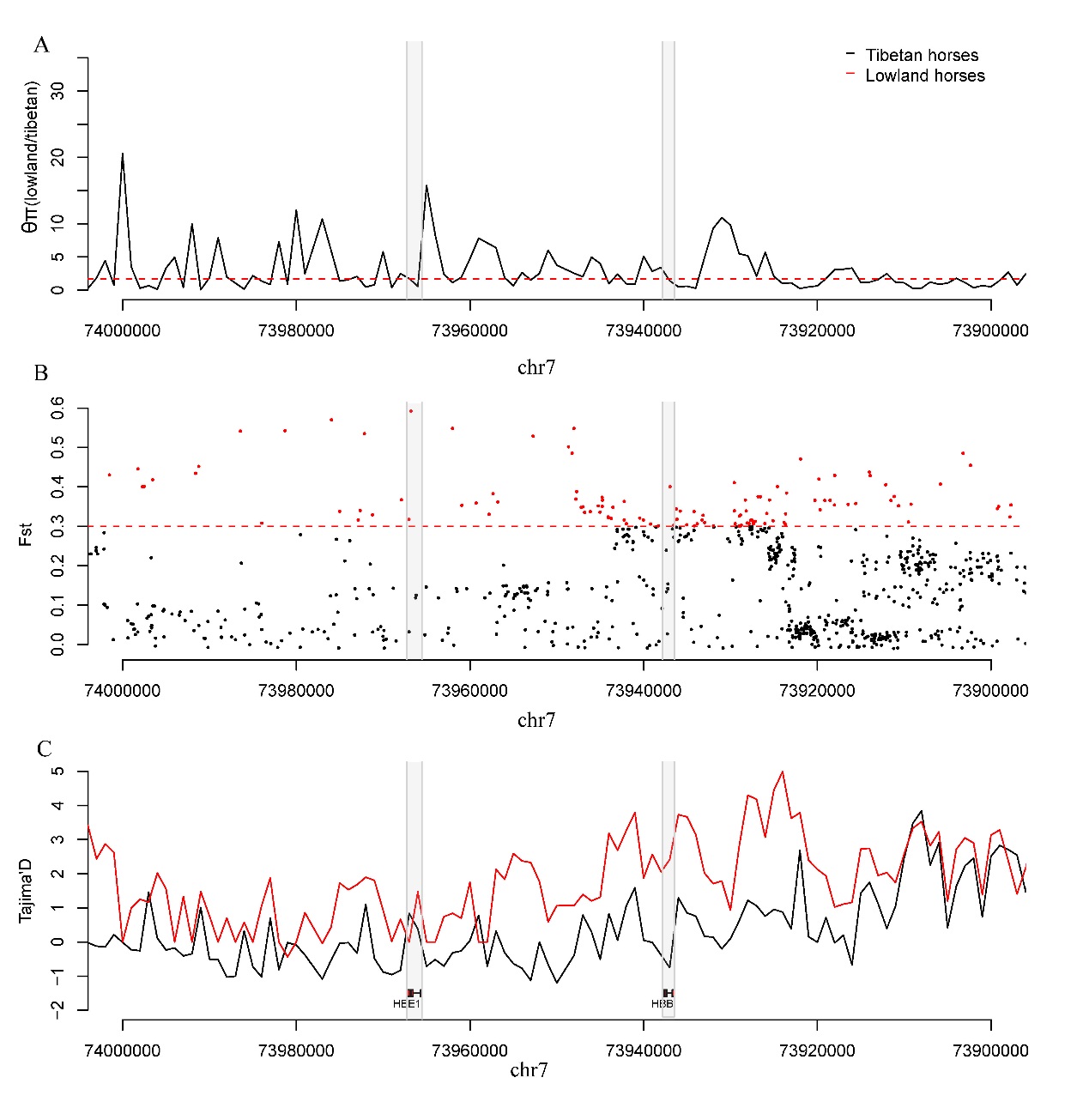


**Figure S11 *HBB* and *HBE1* shows different genetic signatures in horse high altitude adaptation.** The θ_π_ ratio (θπ-_LL_/θπ-_QT_) (**A**) Tajima’s D (**B**) and F_ST_ value (**C**), are plotted against the peak position from 74.0 Mb to 73.9 Mb on chromosome 7. Both θ_π_ ratio and Tajima’s D values were based on a 20 Kb window and a 20 Kb step. The black and the red lines represent the Tajima’s D values for high-altitude and lowland horses, respectively. The gray columns represent the strongest positive selection signatures in the region considered. The small black boxes and short lines represent the gene structure of *HBB* and *HBE1* within the strongest selective signal. The red dot represents the significant threshold of F_ST_ value per SNP greater than 0.3. The top SNP was noted by black arrow.

**
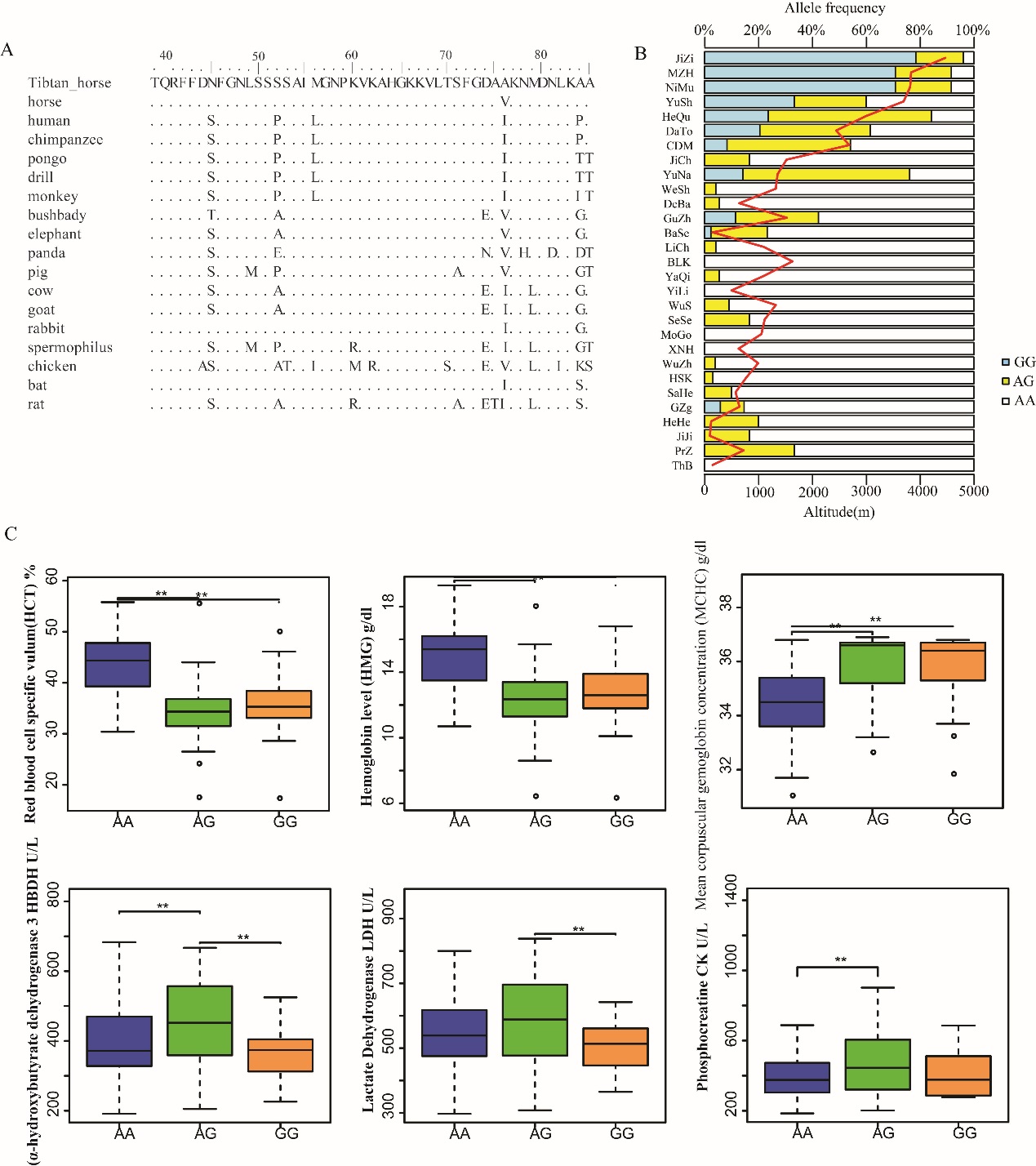
**

**Figure S12 Annotation and validation of the V76A missense SNP under positive selection in the *HBE1*.** (A) HBE1 protein sequence analysis. The protein coordinates are based on the ENSECAT00000015683.1 Ensembl protein. The upper panel shows the Pfam domains of the HBE1 protein. The yellow box represents the hemoglobin domain. The orthologous protein sequences from 17 vertebrates are aligned with the mutant residues shown in the box. horse, ENSECAG00000012269; human, ENSG00000213931; chimpanzee, ENSPTRG00000022526; pongo, ENSPPYG00000003579; drill, ENSMLEG00000032657; monkey, P51440 (uniprot); bushbady, ENSOGAG00000029742; elephant, ENSLAFG00000020785; panda, ENSAMEG00000013439; pig, ENSSSCG00000014726; cow, ENSBTAG00000037815; goat, ENSCHIG00000024977; rabbit, ENSOCUG00000027533; spermophilus, ENSSTOG00000020904; chicken, ENSGALG00000028273; bat, ENSMLUG00000023161; rat, ENSRNOG00000029286. (B) The spectrum of allele frequencies. Genotypes were determined by KASP technology at the missense polymorphism of *HBE1* in the 29 Chinese native horse breeds (N = 908 horses). The altitude information is shown in red line. (C) Association analysis of the *HBE1* SNP (mutant allele, G; reference allele, A) with the individual hemoglobin level and blood physiological values in Hequ and GZg horses. The ANOVA F-test was performed, and we found a significant association between the genotypes and HCT, HMG, MCHC, LDH, HBDH and CK. ** and * displayed the statistical significance of P-value <0.01 and 0.05 according to the ANOVA F-test.


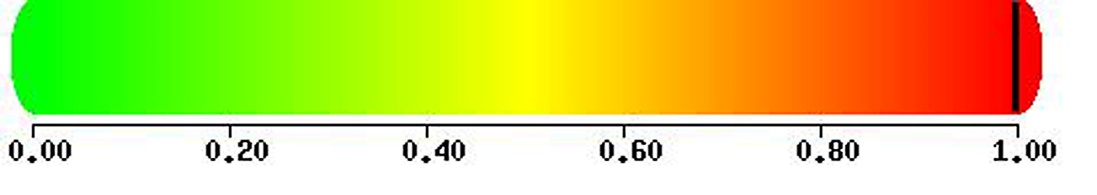

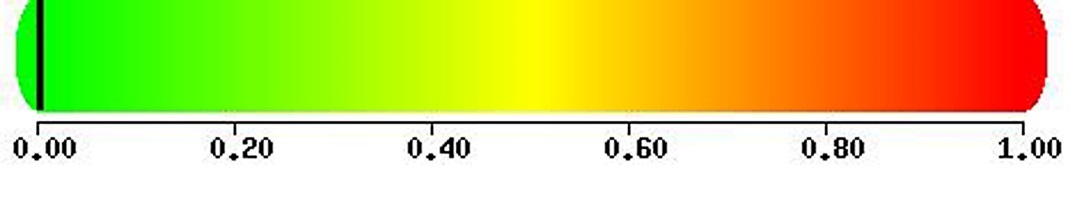


A

B

**Figure S13 Prediction of the functional effects of human nsSNPs (Polyphen2) prediction of the two mutations,** (A) is the *EPAS1-*R144C locus and (B) is the *EPAS1-*E263D locus. 1 means the substitution of animal acid probably damaged the function of protein. 0 means substitution may not change the structure of the protein (Adzhubei et al. 2010).


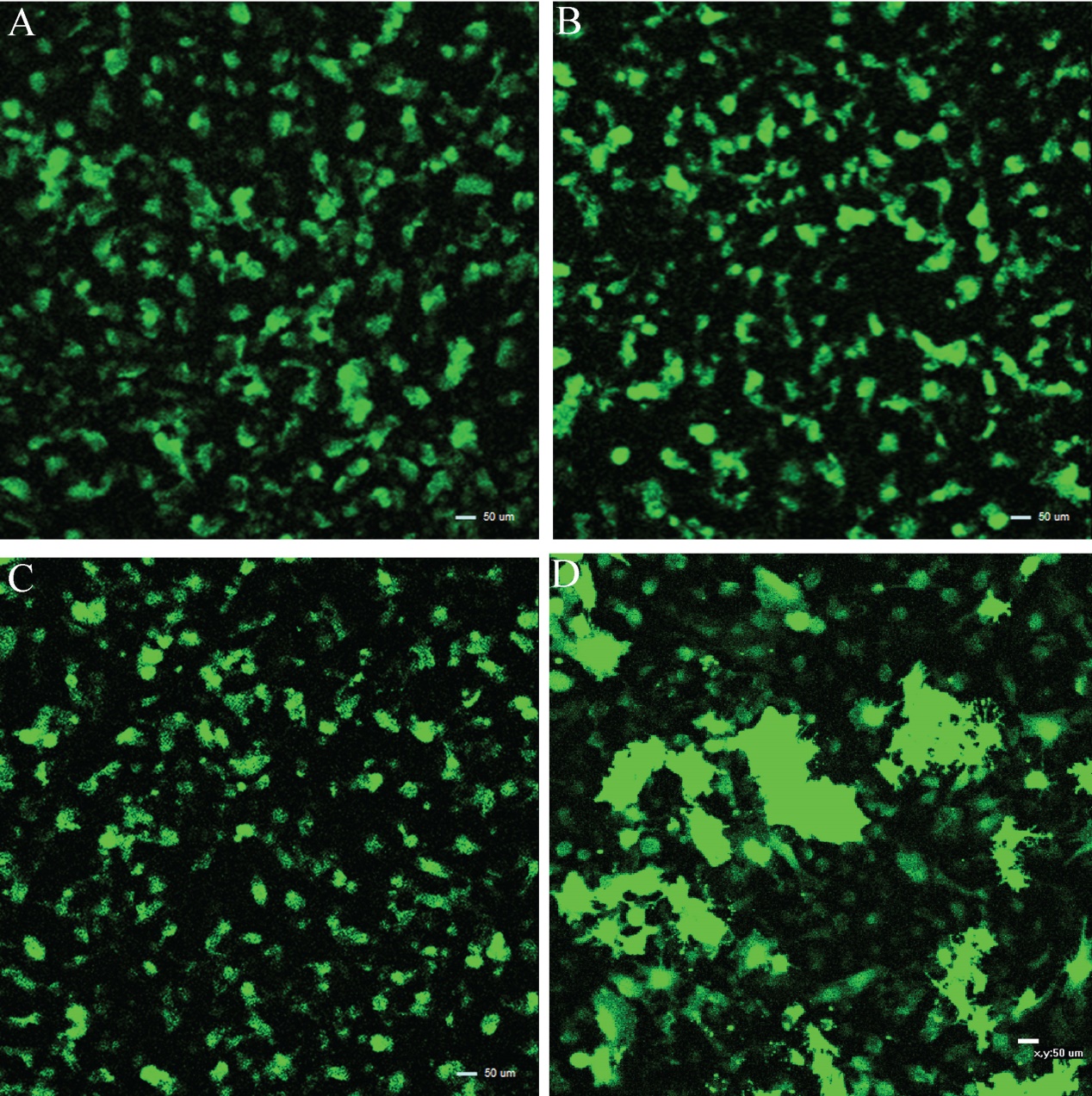


**Figure S14 Plot of A549 cell lysates transfected with the GFP-tagged recombinant plasmid** of WT (A), R144C (B), E263D (C) and the empty vector (D). EPAS1 was a transcription factor that should be expressed in nucleus, while Green fluorescent protein (GFP) was expressed in cytoplasm.

**References**

Adzhubei IA, Schmidt S, Peshkin L, Ramensky VE, Gerasimova A, Bork P, Kondrashov AS, Sunyaev SR. 2010. A method and server for predicting damaging missense mutations. Nat Med 7, 248.

Alexander DH, Novembre J, Lange K. 2009. Fast model-based estimation of ancestry in unrelated individuals. Genom Res 19: 1655-1664.

Beall CM, Cavalleri GL, Deng L, Elston RC, Gao Y, Knight J, Li C, Li JC, Liang Y, McCormack M, et al. 2010. Natural selection on EPAS1 (HIF2α) associated with low hemoglobin concentration in Tibetan highlanders. Proc Natl Acad Sci U S A 107: 11459-11464.

Beall CM, Decker MJ, Brittenham GM, Kushner I, Gebremedhin A, Strohl KP. 2002. An Ethiopian pattern of human adaptation to high-altitude hypoxia. Proc Natl Acad Sci U S A 99: 17215-17218.

Bielejec F, Rambaut A, Suchard MA, Lemey P. 2011. SPREAD: spatial phylogenetic reconstruction of evolutionary dynamics. Bioinformatics 27: 2910-2912.

Bigham AW, Lee FS 2014. Human high-altitude adaptation: forward genetics meets the HIF pathway. Genes Dev 28: 2189-2204.

Cavadas B, Pedro N, Fernandes V, Ferreira JC, Pereira L, Ricaut F-X, Brucato N, Alshamali F. 2019. Genome-Wide Characterization of Arabian Peninsula Populations: Shedding Light on the History of a Fundamental Bridge between Continents. Mol Biol Evol 36: 575-586.

Chen FH, Dong GH, Zhang DJ, Liu XY, Jia X, An CB, Ma MM, Xie YW, Barton L, Ren XY, et al. 2015. Agriculture facilitated permanent human occupation of the Tibetan Plateau after 3600 B.P. Science 347: 248.

Danecek P, Auton A, Abecasis G, Albers CA, Banks E, DePristo MA, Handsaker RE, Lunter G, Marth GT, Sherry ST, et al. 2011. The variant call format and VCFtools. Bioinformatics 27: 2156-2158.

Danecek P, Auton A, Abecasis G, Albers CA, Banks E, DePristo MA, Handsaker RE, Lunter G, Marth GT, et al. 2011b. The variant call format and VCFtools. Bioinformatics 27: 2156-2158.

Drummond AJ, Rambaut A. 2007. BEAST: Bayesian evolutionary analysis by sampling trees. BMC Evol Biol 7: 214.

Excoffier L, Foll M. 2011. fastsimcoal: a continuous-time coalescent simulator of genomic diversity under arbitrarily complex evolutionary scenarios. Bioinformatics 27: 1332-1334.

Gaunitz C, Fages A, Hanghøj K, Albrechtsen A, Khan N, Schubert M, Seguin-Orlando A, Owens IJ, Felkel S, Bignon-Lau O,et al.2018. Ancient genomes revisit the ancestry of domestic and Przewalski’s horses. Science 360: 111.

Ge R-L, Cai Q, Shen Y-Y, San A, Ma L, Zhang Y, Yi X, Chen Y, Yang L, Huang Y, et al.2013. Draft genome sequence of the Tibetan antelope. Nat Commun 4: 1858.

Gou X, Wang Z, Li N, Qiu F, Xu Z, Yan D, Yang S, Jia J, Kong X, Wei Z, et al.2014. Whole-genome sequencing of six dog breeds from continuous altitudes reveals adaptation to high-altitude hypoxia. Genom Res 24: 1308-1315.

Gutenkunst RN, Hernandez RD, Williamson SH, Bustamante CD. 2009. Inferring the Joint Demographic History of Multiple Populations from Multidimensional SNP Frequency Data. PLOS Genet 5: e1000695.

Hendrickson SL. 2013. A genome wide study of genetic adaptation to high altitude in feral Andean Horses of the páramo. BMC Evol Biol 13: 273.

Hoit BD, Dalton ND, Erzurum SC, Laskowski D, Strohl KP, Beall CM. 2005. Nitric oxide and cardiopulmonary hemodynamics in Tibetan highlanders. Journal of Applied Physiology 99: 1796-1801.

Horscroft JA, Kotwica AO, Laner V, West JA, Hennis PJ, Levett DZH, Howard DJ, Fernandez BO, Burgess SL, Ament Z, et al.2017. Metabolic basis to Sherpa altitude adaptation. Proc Natl Acad Sci U S A 114: 6382-6387.

Lee JS, Kim Y, Bhin J, Shin H-JR, Nam HJ, Lee SH, Yoon J-B, Binda O, Gozani O, Hwang D, et al.2011. Hypoxia-induced methylation of a pontin chromatin remodeling factor. ProcNatl Acad Sci U S A 108: 13510-13515.

Li H, Handsaker B, Wysoker A, Fennell T, Ruan J, Homer N, Marth G, Abecasis G, Durbin R. 2009. The Sequence Alignment/Map format and SAMtools. Bioinformatics 25: 2078-2079.

Ling YH, Ma YH, Guan WJ, Cheng YJ, Wang YP, Han JL, Mang L, Zhao QJ, He XH, Pu YB, et al.2011. Evaluation of the genetic diversity and population structure of Chinese indigenous horse breeds using 27 microsatellite markers. Anim Genet 42: 56-65.

Liu X, Huang M, Fan B, Buckler ES, Zhang Z. 2016. Iterative Usage of Fixed and Random Effect Models for Powerful and Efficient Genome-Wide Association Studies. PLOS Genet 12: e1005767.

Lorenzo FR, Huff C, Myllymaki M, Olenchock B, Swierczek S, Tashi T, Gordeuk V, Wuren T, Ri-Li G, McClain DA, et al.2014. A genetic mechanism for Tibetan high-altitude adaptation. Nat Genet 46: 951-956.

Luo JC, Shibuya M. 2001. A variant of nuclear localization signal of bipartite-type is required for the nuclear translocation of hypoxia inducible factors (1α, 2α and 3α). Oncogene 20: 1435.

McCormick RF, Truong SK, Mullet JE. 2015. RIG: Recalibration and Interrelation of Genomic Sequence Data with the GATK. G3-Genes Genom Genet 5: 655.

Nei M, Li WH. 1979. Mathematical model for studying genetic variation in terms of restriction endonucleases. Proc Natl Acad Sci U S A 76: 5269-5273.

Orlando L, Ginolhac A, Zhang G, Froese D, Albrechtsen A, Stiller M, Schubert M, Cappellini E, Petersen B, Moltke I, et al. 2013. Recalibrating Equus evolution using the genome sequence of an early Middle Pleistocene horse. Nature 499: 74.

Pickrell, J. K. , Pritchard, J. K.. 2012. Inference of Population Splits and Mixtures from Genome-Wide Allele Frequency Data. PLOS Genet 8: e1002967.

Prabhakar NR, Semenza GL. 2012. Adaptive and maladaptive cardiorespiratory responses to continuous and intermittent hypoxia mediated by hypoxia-inducible factors 1 and 2. Physiol Rev 92: 967-1003.

Qiu Q, Zhang G, Ma T, Qian W, Wang J, Ye Z, Cao C, Hu Q, Kim J, Larkin DM,et al.2012. The yak genome and adaptation to life at high altitude. Nat Genet 44: 946-949.

Rubin CJ, Megens HJ, Martinez Barrio A, Maqbool K, Sayyab S, Schwochow D, Wang C, Carlborg O, Jern P, Jørgensen CB, et al. 2012. Strong signatures of selection in the domestic pig genome. Proc Natl Acad Sci U S A 109: 19529-19536.

Saitou N, Nei M. 1987. The neighbor-joining method: a new method for reconstructing phylogenetic trees. Mol Biol Evol 4: 406-425.

Sanchez-Mazas A. 2012. Past human migrations in East Asia : matching archaeology, linguistics and genetics. London: Routledge.

Sato M, Tanaka T, Maeno T, Sando Y, Suga T, Maeno Y, Sato H, Nagai R, Kurabayashi M. 2002. Inducible expression of endothelial PAS domain protein-1 by hypoxia in human lung adenocarcinoma A549 cells. Role of Src family kinases-dependent pathway. Am J Respir Cell Mol Biol 26: 127-134.

Semagn K, Babu R, Hearne S, Olsen M. 2014. Single nucleotide polymorphism genotyping using Kompetitive Allele Specific PCR (KASP): overview of the technology and its application in crop improvement. Mol Breeding 33: 1-14.

Simonson TS, Yang Y, Huff CD, Yun H, Qin G, Witherspoon DJ, Bai Z, Lorenzo FR, Xing J, Jorde LB, et al. 2010. Genetic evidence for high-altitude adaptation in Tibet. Science 329 (5987):72-5.

Song S, Yao N, Yang M, Liu X, Dong K, Zhao Q, Pu Y, He X, Guan W, Yang N, et al.2016. Exome sequencing reveals genetic differentiation due to high-altitude adaptation in the Tibetan cashmere goat (Capra hircus). BMC Genomics 17: 122.

Tamura K, Peterson D, Peterson N, Stecher G, Nei M, Kumar S. 2011. MEGA5: molecular evolutionary genetics analysis using maximum likelihood, evolutionary distance, and maximum parsimony methods. Mol Biol Evol 28: 2731-2739.

Velie BD, Fegraeus KJ, Solé M, Rosengren MK, Røed KH, Ihler C-F, Strand E, Lindgren G. 2018. A genome-wide association study for harness racing success in the Norwegian-Swedish coldblooded trotter reveals genes for learning and energy metabolism. BMC Genet 19: 80.

Wade CM, Giulotto E, Sigurdsson S, Zoli M, Gnerre S, Imsland F, Lear TL, Adelson DL, Bailey E, Bellone RR, et al.2009. Genome sequence, comparative analysis, and population genetics of the domestic horse. Science 326: 865-867.

Wang G-D, Fan R-X, Zhai W, Liu F, Wang L, Zhong L, Wu H, Yang H-C, [Wu SF](https://www.ncbi.nlm.nih.gov/pubmed/?term=Wu%20SF%5BAuthor%5D&cauthor=true&cauthor_uid=25091388), [Zhu CL](https://www.ncbi.nlm.nih.gov/pubmed/?term=Zhu%20CL%5BAuthor%5D&cauthor=true&cauthor_uid=25091388), et al.2014. Genetic Convergence in the Adaptation of Dogs and Humans to the High-Altitude Environment of the Tibetan Plateau. Genom Biol Evol 6: 2122-2128.

Wang MS, Li Y, Peng MS, Zhong L, Wang ZJ, Li QY, Tu XL, Dong Y, Zhu CL, Wang L, et al.2015. Genomic Analyses Reveal Potential Independent Adaptation to High Altitude in Tibetan Chickens. Mol Biol Evol 32: 1880-1889.

Wang Q, Li Q, Liu R, Zheng M, Wen J, Zhao G. 2016. Host cell interactome of PA protein of H5N1 influenza A virus in chicken cells. J Proteomics 136: 48-54.

Wei C, Wang H, Liu G, Zhao F, Kijas JW, Ma Y, Lu J, Zhang L, Cao J, Wu M, et al.2016. Genome-wide analysis reveals adaptation to high altitudes in Tibetan sheep. Sci Rep 6: 26770.

Weir BS, Cockerham CC. 1984. Estimating F-Statistics for the Analysis of Population Structure. Evolution 38: 1358-1370.

Xu S, Li S, Yang Y, Tan J, Lou H, Jin W, Yang L, Pan X, Wang J, Shen Y, et al.2011. A genome-wide search for signals of high-altitude adaptation in Tibetans. Mol Biol Evol 28(2):1003-11.

Yang F 2004. The “Ancient Tea and Horse Caravan Road”, the “Silk Road” of Southwest China. The Silk Road 2: 29-33.

Yang J, Jin ZB, Chen J, Huang XF, Li XM, Liang YB, Mao JY, Chen X, Zheng Z, Bakshi A, et al.2017. Genetic signatures of high-altitude adaptation in Tibetans. Proc Natl Acad Sci U S A 114: 4189-4194.

Yi X, Liang Y, Huerta-Sanchez E, Jin X, Cuo ZX, Pool JE, Xu X, Jiang H, Vinckenbosch N, Korneliussen TS, et al.2014. Hypoxia Adaptations in the Grey Wolf (Canis lupus chanco) from Qinghai-Tibet Plateau. PLOS Genet 10: e1004466.

Zhang Z, Jia Y, Almeida P, Mank JE, van Tuinen M, Wang Q, Jiang Z, Chen Y, Zhan K, Hou S, et al.2018. Whole-genome resequencing reveals signatures of selection and timing of duck domestication. Gigascience 7:1-11.

Zhang Z, Xu D, Wang L, Hao J, Wang J, Zhou X, Wang W, Qiu Q, Huang X, Zhou J, et al.2016. Convergent Evolution of Rumen Microbiomes in High-Altitude Mammals. Cur Biol 26: 1873-1879.

Zhou Z, Jiang Y, Wang Z, Gou Z, Lyu J, Li W, Yu Y, Shu L, Zhao Y, Ma Y,et al.2015. Resequencing 302 wild and cultivated accessions identifies genes related to domestication and improvement in soybean. Nat Biot 33: 408-414.
